# Effect of the Substitution Position on the Electronic and Solvatochromic Properties of Isocyanoaminonaphthalene (ICAN) Fluorophores

**DOI:** 10.3390/molecules24132434

**Published:** 2019-07-02

**Authors:** Sándor Lajos Kovács, Miklós Nagy, Péter Pál Fehér, Miklós Zsuga, Sándor Kéki

**Affiliations:** 1 Department of Applied Chemistry, University of Debrecen, Egyetem tér 1., H-4032 Debrecen, Hungary; 2 Institute of Organic Chemistry, Research Centre for Natural Sciences, Hungarian Academy of Sciences, Magyar tudósok körútja 2., H-1519 Budapest, Hungary

**Keywords:** isocyano-aminonaphthalenes, solvatochromism, fluorescence, electronic absorption, push-pull dyes

## Abstract

The properties of 1,4-isocyanoaminonaphthalene (1,4-ICAN) and 2,6-isocyanoaminonaphthalene (2,6-ICAN) isomers are discussed in comparison with those of 1,5-isocyanoaminonaphthalene (1,5-ICAN), which exhibits a large positive solvatochromic shift similar to that of Prodan. In these isocyanoaminonaphthalene derivatives, the isocyano and the amine group serve as the donor and acceptor moieties, respectively. It was found that the positions of the donor and the acceptor groups in these naphthalene derivatives greatly influence the Stokes and solvatochromic shifts, which decrease in the following order: 1,5-ICAN > 2,6-ICAN > 1,4-ICAN. According to high-level quantum chemical calculations, this order is well correlated with the charge transfer character of these compounds upon excitation. Furthermore, unlike 1,5-ICAN, the 1,4-ICAN and 2,6-ICAN isomers showed relatively high quantum yields in water, that were determined to be 0.62 and 0.21, respectively. In addition, time-resolved fluorescence experiments revealed that both the radiative and non-radiative decay rates for these three ICAN isomers varied unusually with the solvent polarity parameter E_T_(30). The explanations of the influence of the solvent polarity on the resulting steady-state and time-resolved fluorescence emission spectra are also discussed.

## 1. Introduction

**Scheme 1:**
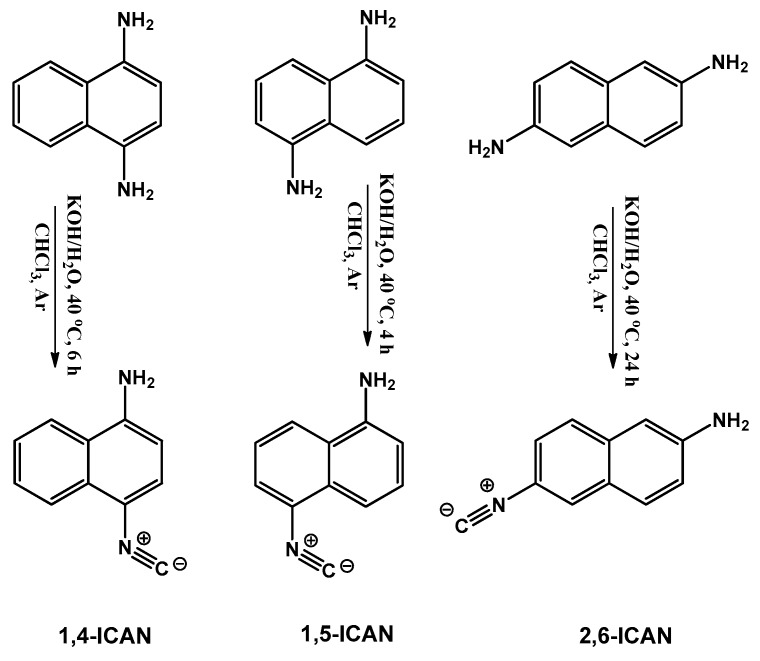
The structures of the isocyanoaminonaphthalene (ICAN) derivatives and their general preparation method.

“Smart” fluorophores, whose emission properties, e.g., the color and the intensity of the emitted light varying with the polarity [1,2,3] or the viscosity of their microenvironment [4], have attracted considerable attention due to their fast and non-destructive abilities to detect changes in their local environment. Thus, the utility of such fluorophores ranges from their applications as probes for the determination of critical micelle concentrations (cmc) of various surfactants [5] through viscosity measurements of biological membranes [6,7] to monitoring the interactions of protein bindings [8,9]. Fluorophores, whose fluorescence emissions are particularly sensitive to the polarity of their microenvironment alter the emitted light color upon the effect of polarity change, and are hence called solvatochromic fluorophores [10,11]. Such molecules are typically built up from an electron-donating (donor, D) and an electron-withdrawing (acceptor, A) group, connected through an aromatic π-linker moiety, which is usually naphthalene or a fluorene ring-system. These so-called D-π-A type solvatochromic fluorophores are, therefore, based on the shift of the electron density from the donor group to the acceptor moiety through the π-system upon excitation; hence, an intramolecular charge-transfer (ICT) takes place [10,11,12,13,14]. Furthermore, ICT may give rise to an increase in the excited dipole moment state with respect to that of the ground state. It is believed that the presence of ICT, in the absence of any specific interaction, i.e., hydrogen-bonding, between the fluorophore and the solvent, is the primary reason for the increasing solvatochromic and Stokes shifts with solvent polarity. Until now, several “push-pull” D-π-A type solvatochromic fluorophores have been synthesized. The seminal and the most prominent members of this fluorophore family displaying notable solvatochromism are the 2-(dimethylamino)-6-propionylnaphthalene, also known as Prodan and its related derivatives [2]. Recently, we have synthesized a series of new solvatochromic fluorophores based on the preliminary concept of ICT in which the donor amine and the acceptor isocyano groups are connected via the naphthalene moiety in its 1,5-positions to yield 1,5-isocyanoamino-(1,5-ICAN) derivatives [15,16,17,18]. The 1,5-ICAN exhibits large solvatochromic and Stokes shifts similar to the Prodan [15]. Despite their very simple structure, few members of the isocyanoaminoarene group have been prepared and studied until recently, most probably due to the infamous putrid odor of the common isocyano compounds. Nevertheless, none of the ICAN derivatives prepared by us have any odor at all. On the contrary, they proved to be one of the most versatile “smart” fluorophore dye families, which found applications as nontoxic supravital stains for the investigation and characterization of both plant and animal cells [19,20], enable the selective detection of Hg^2+^, and at the same time are able to indicate the presence of Ag^+^, which is unprecedented among fluorescent sensors [21]. In addition, to the best of our knowledge, ICAN is the simplest and lowest molecular weight fluorophore that has ever been used for fluorescent Hg(II) detection. ICANs can also be utilized in silver analytics as isocyanide ligands, and their silver complexes turned out to enhance the contrast during cell staining applications [18]. Furthermore, substitutions of the hydrogen atoms of the amino group in the 1,5-ICAN derivative with methyl-, allyl- or acryl groups have made the 1,5-ICAN more appropriate as vital fluorescent probes [17,19]. The field of isocyanoaminoarenes is still unexplored; however, we believe that they may contain the considerable potential to become versatile smart dyes. However, the rational design and development of more efficient solvatochromic fluorophores require a deeper understanding of their structure-property relationships. One of the key factors, which have not been explored yet in connection with these ICAN fluorophores, is the effect of the substitution position of the amino and the isocyano groups. Thus, in this article, the electronic, solvatochromic and the photophysical properties of the novel 1-amino-4-isocyanonaphthalene (1,4-ICAN) and the Prodan analog, 2-amino-6-isocyanonaphthalene (2,6-ICAN) are reported and compared to those obtained for the 1,5-ICAN isomer (Scheme 1).

## 2. Results and Discussion

### 2.1. UV-Vis Electronic Absorption Properties of ICAN Isomers

Recently, we reported the electronic absorption and the fluorescence emission properties of the 1,5-ICAN isomer and the corresponding data are found in ref [15]. For the sake of better comparison of its behavior with those of the other ICAN isomers, some representative data are inserted in the following.

**Figure 1:**
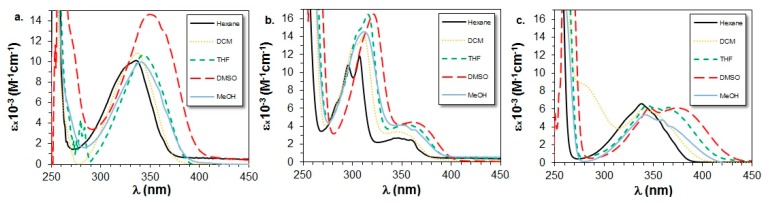
The UV-Vis spectra of the 1,4-ICAN (**a**), 2,6-ICAN (**b**), and 1,5-ICAN (**c**) isomers recorded in different solvents.

In order to learn more about the ground state electronic properties of the 1,4- and 2,6-ICAN isomers, they were dissolved in various solvents, and their UV-Vis spectra were recorded in the wavelength range of 200 nm to 700 nm. The solvents were chosen to cover a broad range of solvent polarity, spanning from the non-polar hexane to the polar DMSO. The UV-Vis spectra of 1,4-ICAN and 2,6-ICAN along with 1,5-ICAN in different solvents are shown in Figure 1, while the absorption maximum wavelength (λ_Abs_) and the corresponding molar absorption coefficients (ε) are compiled in Table 1.

As seen in Figure 1, in the wavelength range of 300–400 nm which belongs to the lowest electronic energy transition for the ICAN isomers, the UV-Vis spectrum of 1,4-ICAN shows only a single band centered at around 350 nm. In contrast, the 2,6-ICAN isomer displays two markedly different bands in this wavelength region, the first band centered at around 320 nm while the second one occurs at approximately 350 nm. It should be noted that the UV-Vis spectrum of 1,5-ICAN is rather similar to that of the 2,6-ICAN. Furthermore, it can also be observed that the lowest electronic transitions at *ca* 350 nm occur with significantly higher ε values for 1,4-ICAN than for the 2,6-ICAN and the 1,5-ICAN isomers as confirmed by Figure 1 and the data in Table 1. In addition, DFT-calculations, in line with the experimental observations, also revealed that the oscillator strength (i.e., ε values) increased in the order of 2,6-ICAN < 1,5-ICAN < 1,4-ICAN. In relation to the dependence of λ_Abs_ on the solvent polarity, it could also be surmised from the data of Table 1 that the absorption bands at around 350 nm suffer from slight red-shifts with increasing solvent polarity. For example, going from n-hexane to DMSO, the bathochromic shifts are 9 nm, 24 nm, and 12 nm for the 1,5-ICAN, 1,4-ICAN, and 2,6-ICAN isomers, respectively. In addition, the values of λ_Abs_ for the 1,5-ICAN and 1,4-ICAN isomers are very similar, whereas the λ_Abs_ occur at longer wavelengths for the 2,6-ICAN isomer in all the solvents except DMF and DMSO, where λ_Abs_ values follow the order: 1,5-ICAN < 1,4-ICAN < 2,6-ICAN.

The shifts of the low energy bands are indicative of the polar character of the ground state. Indeed, DFT-calculations yielded 7.2 D, 8.0 D, and 8.6 D ground state dipole moments for the 1,5-ICAN, 1,4-ICAN, and 2,6-ICAN isomers, respectively. Interestingly, λ_Abs_ values in water, as in the most polar compound listed in Table 1, is lower than those measured in DMSO for all the three ICAN isomers. The wavelength differences (λ_Abs,DMSO–_λ_Abs,H2O_) are 11 nm, 26 nm, and 24 nm for the 1,5-ICAN, 1,4-ICAN, and 2,6-ICAN, respectively. The reason for this finding may be that the amino group of these isomers, due to the formation of hydrogen bonds, strongly interacts with the water molecules as we have previously shown for pyridine [16]. This interaction reduces the electron density on the N-atom of the amino moiety giving rise to a hypsochromic shift with respect to DMSO.

**Figure 2:**
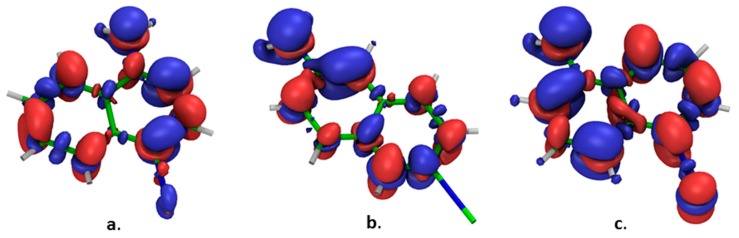
Calculated electronic density differences between the first vertical excited state and the ground state in DMSO for the 1,4-ICAN (**a**), 2,6-ICAN (**b**), and 1,5-ICAN (**c**) isomers.

To better describe the excitation behavior of the ICAN isomers, we have calculated their electronic density differences between the first vertical excited state and the ground state in DMSO as represented in Figure 2.

We have chosen DMSO because it is the most polar nonprotic solvent that should show the most pronounced effect on the transition. It can be seen clearly that the blue areas, indicating the loss of electronic density, are located on the amino nitrogen and the near carbons in each isomer, while the red areas, showing the increase of electronic density, are different for 1,5-ICAN and the other two derivates. For 1,5-ICAN, this region is located on the far ring and the isocyano moiety, while the other two show nearly negligible change on the isocyano group. This suggests that although charge gets transferred from the amino group in all cases, it only reaches the desired acceptor in case of the 1,5-ICAN, while the other two exhibit local changes in the naphthalene ring. Therefore, the proper ICT character can only be attributed to the 1,5-ICAN according to this model.

### 2.2. Steady-State Fluorescence Emission Properties of ICAN Isomers

**Figure 3:**
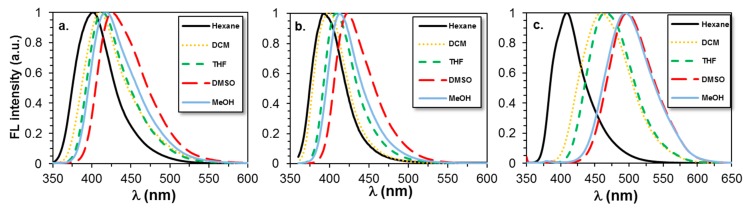
Normalized fluorescence emission spectra of the 1,4-ICAN (**a**), 2,6-ICAN (**b**), and 1,5-ICAN (**c**) isomers recorded in different solvents.

The normalized fluorescence emission spectra for the 1,4-ICAN and 2,6-ICAN isomers are shown in Figure 3, and the fluorescence emission maxima (λ_Em_), and the quantum yields (Φ_f_) determined in various solvents for the 1,5-ICAN, 1,4-ICAN, and 2,6-ICAN isomers are summarized in Table 2.

As seen in Figure 3, the fluorescence emission spectra of both 1,4-ICAN and 2,6-ICAN in various solvents show unstructured, single bands, similar to those of 1,5-ICAN [15], revealing positive solvatochromic shifts upon increasing the solvent polarity from n-hexane to DMSO. In addition, the same is valid for the fluorescence emission bandwidth at half maximum (Δν_1/2_), which may be indicative of the extent of intramolecular charge transfer (ICT) upon excitation. For example, in dioxane, the Δν_1/2_ increases in the order of 2,6-ICAN (2660 cm^−1^) < 1,4-ICAN (3220 cm^−1^) < 1,5-ICAN (3625 cm^−1^). Stemming from the data of Table 2, it could also be realized that the fluorescence emission maxima in all the solvents follow the same order, i.e., the λ_Em_ increases in the order of 2,6-ICAN < 1,4-ICAN < 1,5-ICAN in good agreement with the TD-DFT calculations as illustrated in Figure S13 in the Supporting Information.

According to the data of Table 2, the solvatochromic shifts from hexane to DMSO are 88 nm, 23 nm, and 25 nm for the 1,5-ICAN, 1,4-ICAN, and 2,6-ICAN, respectively. The relatively small solvatochromic shift for the 2,6-ICAN isomer is surprising in light of the fact that the longest dipole distance between the donor amino and the acceptor isocyano group is available in the 2,6-ICAN isomer, as will be shown later. As a consequence, this finding may shed light also on the fact that a rational design of effective fluorophores which requires extensive prior knowledge on the electronic absorption and fluorescence emission properties of a specific class of compounds to be developed. Furthermore, the data in Table 2 also indicate that the quantum yields (Φ_f_) vary significantly with the solvent used. The highest Φ_f_ were obtained for the 1,5-ICAN (0.28–0.95), whereas 1,4-ICAN isomer yields lower Φ_f_ values (0.41–0.86), and the lowest Φ_f_ values were determined in the case of the 2,6-ICAN isomer (0.15–0.53). It is also intriguing that the highest Φ_f_ were found in dioxane for the 1,5-ICAN (0.95) and the 1,4-ICAN (0.86), while the 2,6-ICAN isomer displays the highest Φ_f_ in highly polar solvents, such as DMF (0.53) and DMSO (0.51). In addition, it is to be noted that relatively high Φ_f_ values were found for all the three ICAN isomers in highly polar solvents indicating that the fluorescence decay by a non-radiative triplet-singlet transition does not play a significant role, as Nad et al. pointed out for Coumarin-151 [22], which is very similar to our ICAN dyes. Another interesting finding is the low Φ_f_ value for the 1,5-ICAN (0.04), while appreciable Φ_f_ values were found for the 1,4-ICAN (0.63) and 2,6-ICAN (0.21) isomers in water. An explanation for this finding will be given later.

**Figure 4:**
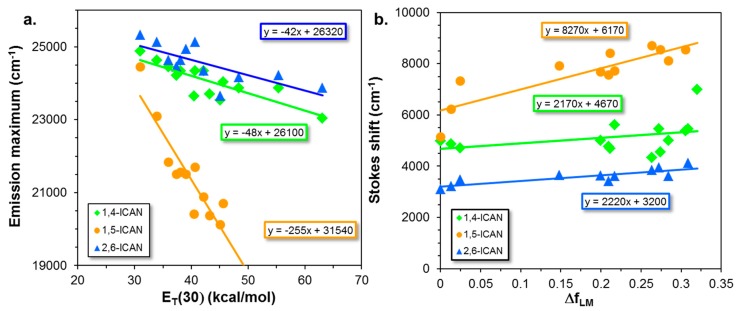
Variation of the fluorescence emission maximum with the empirical solvent polarity parameter E_T_(30) (**a**), and the Lippert–Mataga (LM) (**b**), plots for the 1,4-ICAN, 1,5-ICAN, and the 2,6-ICAN isomers. The data for the 1,5-ICAN isomer are taken from ref [15].

In order to quantify the solvatochromic effect induced by the solvents of different polarity, the fluorescence emission maxima (ν_Em_) are plotted as a function of the empirical solvent polarity parameter E_T_(30) [23] and the Lippert–Mataga (LM) plot [24,25], i.e., a plot for the Stokes shifts (Δν_SS_) versus the orientation polarizations (Δf_LM_) are constructed and presented in Figure 4.

As it turns out from Figure 4a, there are linear correlations between the fluorescence emission maximum and E_T_(30) for all the three fluorophores. On the other hand, as judging from the corresponding slopes illustrated in Figure 4a, it is also evident that the highest solvatochromic shift, i.e., the highest slope can be observed for the 1,5-ICAN isomer (255 kcal^−1^cm^−1^mol), while these values are very similar for the 1,4-ICAN (48 kcal^−1^cm^−1^mol) and the 2,6-ICAN (42 kcal^−1^cm^−1^mol) isomers. This reflects the computational findings presented in Figure 2. To gain quantitative insight, the Lippert–Mataga equation (Equation (1)) can be employed, although it should be noted that the model has some inherent restrictions. Nevertheless, it provides a way to calculate the difference of the excited (µ_E_) and the ground state (µ_G_) dipole moments, which is the key factor in determining the Stokes-shift (Δν_SS_) of a fluorophore according to Equation (1).

(1) $$\Delta \nu_{\mathrm{SS}}=\frac{2{\left(\mu_{E}-\mu_{G}\right)}^{2}}{4\pi \varepsilon_{o}{\mathrm{hca}}_{o}^{3}}\Delta f_{\mathrm{LM}}+\mathrm{const}$$

where Δν_SS_ (in cm^−1^) is the Stokes shift, h, c, and ε_o_ are the Planck’s constant, speed of the light in vacuum, and the permittivity of the vacuum, respectively, whereas a_o_ is the Onsager-radius, which closely reflects the radius of a spherical cavity the fluorophore molecule occupies. Δf_LM_ stands for the orientation polarizability defined as:

(2) $$\Delta f_{\mathrm{LM}}=\frac{\varepsilon -1}{2\varepsilon -1}-\frac{n^{2}-1}{{2n}^{2}-1}$$

where ε and n are the dielectric constants and the refractive index of the solvent, respectively.

According to Figure 4b, the 1,5-ICAN isomer yields much higher slope (8270 cm^−1^) obtained from the LM-plot than do the 1,4-ICAN and 2,6-ICAN isomers, whose LM-plots give very similar slopes (2170 and 2220 cm^−1^, respectively). In addition, the Stokes shifts at Δf_LM_ = 0, i.e., the intercepts of the lines determined from the LM-plots decrease in the order of 1,5-ICAN > 1,4-ICAN > 2,6-ICAN. The LM-plot can also be used to estimate the difference in the values of the excited and the ground state dipole moments, i.e., Δµ = µ_E_ − µ_G_ according to Equation (1). The values of a_o_ for the determination of Δμ from the corresponding LM-plots were obtained as the half distance between the amino and isocyano groups of the corresponding DFT-optimized geometries. The values of a_o_, (µ_E_ − µ_G_, calculated for vertical emission) calculated by the DFT and determined from the corresponding LM-plots are listed in Table 3.

As seen from the data in Table 3, the calculated and the determined values of Δµ from the LM-plots agree reasonably well. It should be noted; however, that the value of Δμ, according to Equation (1), markedly depends on the value of a_o_.

It must be noted here that there is a different, i.e., another accepted way to determine a_o_. This is based on the following relationship: $a_{o}={\left[3M/(4\pi N_{A}\rho )\right]}^{1/3}$, where M is the molecular weight of the fluorophore, N_A_ is the Avogadro’s constant, and ρ is the density. For example, for the 1,5-ICAN isomer taking the density 1 g/cm^3^, a value of a_o_ = 400 pm and Δµ = 7.2 D could be calculated [15].

In order to get a deeper insight into the excited state electronic behavior of these ICAN isomers, further TD-DFT calculations were performed. For the calculations of the electronic transitions, both the ground- and excited-state geometries were optimized. The Highest Occupied Molecular Orbital (HOMO), Lowest Unoccupied Molecular Orbital (LUMO), HOMO − 1, and LUMO + 1 molecular orbitals for the 1,4-ICAN, 1,5-ICAN, and 2,6-ICAN isomers are presented in Figure S14. As these orbitals are spread over the whole molecule in each case, they were not analyzed further, so the reader should refer to Figure 2 for the electronic changes accompanying the absorption process.

**Figure 5:**
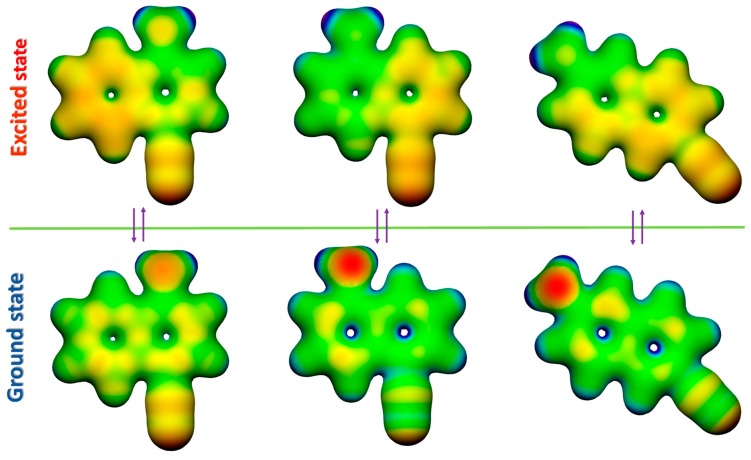
Electrostatic potentials (ESP) for the relaxed ground and excited states of the 1,4-ICAN, 1,5-ICAN, and 2,6-ICAN isomer (from left to right).

Additional information could be obtained on the distribution of the electrons by performing a thorough charge analysis (Tables S3–S5) or mapping the electrostatic potential (ESP) of the relaxed ground and excited states as shown in Figure 5. In every case, a large negative charge is centered on the amino nitrogen, which diminishes upon reaching the relaxed excited state. This is also reflected by a change in the geometry as the amino group flattens, or in other words, rehybridizes to an sp^2^ state where the nonbonding electrons enter the conjugated π system of the naphthalene ring. The isocyano group; however, shows a different behavior for 1,4-ICAN and the other two molecules. In 1,4-ICAN, a considerable negative charge is centered on this moiety in the ground state already, which changes only slightly upon reaching the relaxed excited state. Therefore, in 1,4-ICAN, simply a local excitation from the amino group to the naphthalene ring takes place. The 1,5-ICAN and 2,6-ICAN isomers; however, reveal a charge transfer from the donor amino to the acceptor isocyano group, which is located on the far ring. A comparison to the vertical excitation (Figure 2) reveals that the final electronic configuration of the excited state is reached almost instantaneously after excitation in 1,5-ICAN, while in 2,6-ICAN this requires changes in the geometry as well. Therefore, in 2,6-ICAN, a charge transfer does take place, but in a non-emissive path via excited state relaxation. The vertical emission process from the relaxed excited state (Figure S16) is essentially the reverse of the absorption, indicating that in both processes only the 1,5-ICAN exhibits charge transfer behavior. In 1,4-ICAN, the charge is only locally transferred, while in 2,6-ICAN, an ICT takes place non-radiatively for the whole absorption-emission cycle.

### 2.3. Time-Resolved Fluorescence Measurements

**Figure 6:**
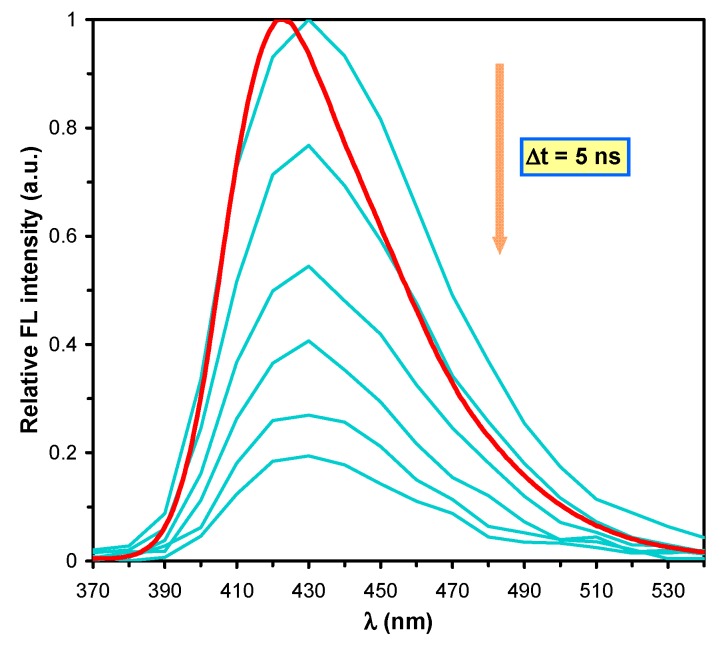
Time-resolved (blue curves) and steady-state emission (red curve) spectra of the 2,6-ICAN isomer in DMSO. The time-resolved emission spectra were recorded at 5 ns intervals.

The time-resolved fluorescence experiments were carried out using laser flash photometry as outlined in the Experimental section. The time-dependent fluorescence emission spectra for the ICAN isomers were recorded in the range from 370 nm to 540 nm in solvents of different polarity in order to shed light on the potential variation of the photophysical parameters with the environment. As an example, the time-resolved fluorescence spectra of the 2,6-ICAN isomer in DMSO are illustrated in Figure 6 (The fluorescence decay curves in different solvents are presented in Figures S17 and S18) For comparison, the steady-state fluorescence emission spectrum is also included.

As seen in Figure 6, the steady-state spectrum matched well with the time-resolved spectra. Furthermore, it is also evident from Figure 6 that there are no shifts in the positions of the time-resolved emission spectra recorded at different times indicating that emissions take place most likely from a fully solvent-relaxed state. From the decay of the fluorescence intensity, the fluorescence decay rate (k_F_), the radiative decay rate (k_r_), and the non-radiative decay rate (k_nr_) were calculated according to Equations (3) and (4).

k_F_ = k_r_ + k_nr_ (3)

k_r_ = Φ_f_k_F_ (4)

On the other hand, it is also possible to calculate the radiative decay rate from the electronic absorption and the fluorescence emission spectra using the Strickler–Berg (SB) equation [26] (Equation (4))

(5) $$k_{\mathrm{SB}}(s^{-1})=2.88\times 10^{-9}n^{2}\frac{\int F(\bar{\nu })d\bar{\nu }}{\int F(\bar{\nu }){\bar{\nu }}^{3}d\bar{\nu }}\int \frac{\varepsilon (\bar{\nu })}{\bar{\nu }}d\bar{\nu }$$

where k_SB_ is the decay rate calculated by the SB equation, n is the refractive index of the solvent, ε($\bar{\nu }$) is the molar absorption coefficient, and F($\bar{\nu }$) is the fluorescence intensity at wave number $\bar{\nu }$.

The values of k_F_, k_r_, k_nr_, and k_SB_ for the three ICAN isomers are compiled in Table 4.

**Figure 7:**
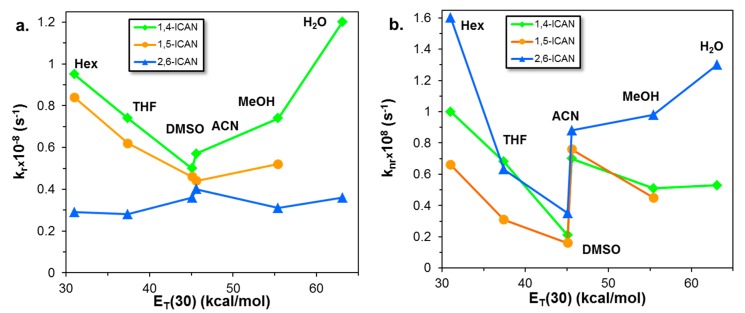
The variation of the radiative decay rate (k_r_) (**a**) and the non-radiative decay rate (k_nr_) (**b**) the with the empirical solvent polarity parameter E_T_(30).

As it turns out from the data in Table 4, the values of k_F_ decrease from hexane to DMSO for all the three ICAN isomers. For example, in the case of 2,6-ICAN, the values of k_F_ are 1.89 × 10^8^ s^−1^ and 7.1 × 10^7^ s^−1^ corresponding to τ = 5.3 ns and 14.1 ns lifetime for the excited state in hexane and the DMSO, respectively. However, in water, the values of k_F_ increase again close to the values measured in hexane. Except for a few cases, the corresponding k_F_ values determined for the three ICAN isomers are very similar to each other; however, the k_r_ and k_nr_ values differ considerably. Furthermore, in spite of the simplicity of the Strickler–Berg equation (Equation (5)), the values of k_SB_ and k_r_ agree relatively well. In order to find out how the solvent polarity influences the values of k_r_ and k_nr_ of the ICAN isomers, k_r_ and k_nr_ are plotted as a function of the empirical solvent polarity parameter E_T_(30), and these plots are shown in Figure 7.

According to Figure 7, both k_r_ and k_nr_ show a minimum value as a function of E_T_(30) for the 1,5-ICAN and 1,4-ICAN isomers in DMSO; however, for the 2,6-ICAN isomer, the values of k_r_ do not change significantly through the entire E_T_(30) range investigated (Figure 7a), whereas k_nr_ versus E_T_(30) goes through minimum values as seen in Figure 7b. The relatively high value of k_nr_ in low polarity solvents (e.g., in n-hexane and THF) may be due to the rotation of the amino group of these ICAN isomers, which initiates a rapid deactivation route from the excited state to the ground state [22]. At higher polarities; however, such a rotation is hindered due to the interaction of the π-system with the amino group that brings about a decrease in the k_nr_ values [22]. In addition, further increases in the solvent polarity resulted in an increase in the values of k_nr_. This finding may be attributed to the increasing ICT character of these ICAN isomers with the solvent polarity and due to the interaction of their amino moiety with the protic solvents (MeOH, H_2_O), which may promote the non-radiative deactivation.

**Figure 8:**
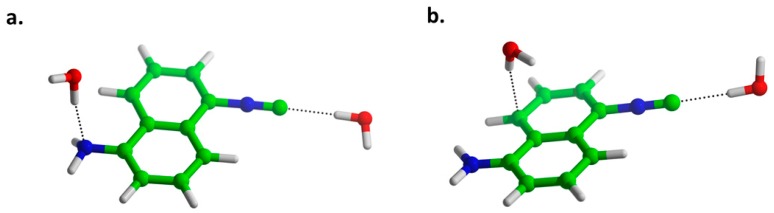
Solvent rearrangement in the ground- (**a**) and in the first relaxed excited state (**b**) for the 1,5-ICAN isomer in water.

To better understand the behavior of the ICAN derivatives in protic solvents, such as water, we have performed calculations where we included two explicit water molecules. One was placed to the amino and the other one to the isocyano group of 1,5-ICAN as shown in Figure 8.

The bulk of the water was modeled as a continuum, using Integral Equation Formalism Polarizable Continuum Model (IEFPCM), like the hexane and DMSO solvents. The optimization of the excited state geometry revealed a rearrangement of the amino hydrogen bond. This behavior is like what was observed in DMSO, which is the shift of the negative charge from the nitrogen to the carbons and the isocyano group as shown in Figure 5. The model, therefore, can capture the specific solvent-solute interaction in protic solvents, which is also indicated by the very accurate characteristic absorption band position at 355 nm (~6% error). In the excited state, both the neutral and protonated (ammonium) forms were optimized. The former yielded an emission band at 537 nm close to the observed wavelength (513 nm), while the latter is a dark state (close to the zero oscillator strength). Although the neutral isomer is more stable in the model, the protonation or a protonation-induced proton exchange might take place as the molecules can overcome energy barriers in the excited state more easily than in the ground state, resulting in fluorescence loss.

## 3. Materials and Methods

### 3.1. Materials

Acetone, dichloromethane (DCM), *n*-hexane, 2-propanol, toluene (reagent grade) were obtained from Molar Chemicals, Hungary, and they were purified by atmospheric pressure distillation. Furthermore, acetonitrile (MeCN), tetrahydrofuran (THF), methanol (MeOH), dimethyl-formamide (DMF), dimethyl-sulfoxide (DMSO), pyridine (HPLC grade, VWR, Darmstadt, Germany), ethylacetate (EtOAc), (reagent grade, Molar Chemicals, Hungary), 1,4-dioxane (reagent grade, Reanal, Hungary), 1,4-diaminonaphthalene (Sigma-Aldrich, Schnelldorf, Germany) were used without further purification. 2,6-diaminonaphthalene was purchased from Manchester Organics (London, UK) and purified by column chromatography.

The 1-amino-4-isocyanonaphthalene (1,4-ICAN) and the 2-amino-6-isocyanonaphthalene (2,6-ICAN) isomers were synthesized from the corresponding diamines (1,4- and 2,6-diaminonaphthalene), and the obtained products were characterized similarly to that reported for the 1-amino-5-isocyanonaphthalene [15]. The details of the synthesis and the characterization of 1,4-ICAN and 2,6-ICAN are given in the Supporting Information.

### 3.2. Instrumental Methods

The UV-Vis spectra were recorded on an Agilent Cary 60 spectrophotometer (Agilent, Santa Clara, CA, USA) spectrophotometer in a quartz cuvette with 1 cm optical length. The volume of the solution was 3.00 cm^3^.

Steady-state fluorescence measurements were performed using a Jasco FP-8200 fluorescence spectrophotometer equipped with a Xe lamp light source. The excitation and emission spectra were recorded at room temperature, using 2.5 nm excitation, 5.0 nm emission bandwidth, and 100 nm/min scanning speed. Fluorescence quantum yields were calculated by using quinine-sulfate in 0.1 mol/L sulfuric acid as the reference absolute quantum yield (Φ_f_ = 0.55).

For UV-Vis and the steady-state fluorescence measurements, ICAN isomers were dissolved in various solvents at a concentration of 0.2 mg/mL (1.19 mM) and diluted to 4.00 mg/L (2.38 × 10^−5^ M) and 0.800 mg/L (4.76 × 10^−6^ M), respectively.

Laser flash photolysis experiments were carried out using an Applied Photophysics (Leatherhead, UK) LKS.60 nanosecond transient absorption spectrometer, equipped with a Quantel Brilliant Nd:YAG laser along with its second, third, and fourth harmonic generator. The third harmonic was used, which emits at 355 nm. For the evaluations of the time-resolved fluorescence curves “DecayFit 1.4”, the time-resolved fluorescence decay fit software downloaded freely from www.fluortools.com was used by applying the tail-fitting method to obtain the corresponding fluorescence decay rates.

### 3.3. Computational Methods

The density functional theory (DFT) calculations were carried out using the Gaussian09 software package [27]. To obtain the ground state properties, the 1,4-ICAN, 1,5-ICAN, and the 2,6-ICAN molecules were optimized in the Polarizable Continuum model (PCM) [28] solvent (hexane and DMSO) cavity using the M06 function [29] together with the Valence Triple-Zeta Polarization (TZVP) basis set [30]. Frequency calculations were done on the optimized structures to verify them as stationary points and obtain Gibbs free energy corrections. The electronic excitations were calculated using the time-dependent density functional theory (TD-DFT) employing the same functional and basis mentioned above. The M06 function was chosen from the tested M06-L, M06, M06-2X, M06-HF [29], and CAM-B3LYP [31] functions because it reproduces the experimental emission wavelengths most accurately (a thorough comparison between M06 and CAM-B3LYP is presented in the Supporting Information in Tables S1 and S2). In addition to modeling the vertical excitation, the molecules were optimized on the potential surface of the first singlet excited state to obtain the excited state properties such as dipole moments and electrostatic potentials. The solvent effects were also included during the optimizations via PCM. To further refine the vertical (de-)excitations, the corrected linear response formalism was employed [32] to properly account for nonequilibrium solvation.

## 4. Conclusions

The electronic absorption, solvatochromic and photophysical properties of three different ICAN isomers including 1,5-ICAN, 1,4-ICAN, and 2,6-ICAN were studied and compared. It was found that the position of the donor amino and the acceptor isocyano group markedly affect these properties. The molar absorption coefficients in various solvents were found to decrease in the order of 1,4-ICAN > 1,5-ICAN > 2,6-ICAN, and this order is in line with the one calculated by the high-level quantum chemical method (DFT). On the other hand, the Stokes shifts (Δν_SS_) for these ICAN isomers were interpreted in terms of the Lippert–Mataga (LM) theory and the values of Δν_SS_ were found to decrease in the order of 1,5-ICAN > 1,4-ICAN ≈ 2,6-ICAN. From the LM-plots, the corresponding differences in the dipole moments of the excited and the ground states were determined, and good agreements with the DFT calculations were obtained. Furthermore, the time-resolved fluorescence emission experiments revealed that (i) emissions take place from the solvent-relaxed state. (ii) both k_r_ and k_nr_ showed a minimum value as a function of the solvent polarity parameter E_T_(30) for the 1,5-ICAN and 1,4-ICAN isomers in DMSO, but for the 2,6-ICAN isomer, k_r_ was found to be nearly constant in the range of the solvent polarity parameter investigated.

In addition, this study may shed light on the fact that a rational design of effective fluorophores requires extensive prior knowledge on the electronic absorption and fluorescence emission properties of the given class of compounds.

## Figures and Tables

**Table 1:** The maximum absorption wavelengths (λ_Abs_) and the molar absorption coefficients at λ_Abs_ (ε) determined in various solvents for the 1,5-ICAN, 1,4-ICAN, and 2,6-ICAN isomers. The dielectric constants of the solvents (ε_r_) are listed next to the solvent names.

	1,5-ICAN *		1,4-ICAN		2,6-ICAN	
Solvent (ε_r_)	λ_Abs_ (nm)	ε (M^−1^cm^−1^)	λ_Abs_ (nm)	ε (M^−1^cm^−1^)	λ_Abs_ (nm)	ε (M^−1^cm^−1^)
*n*-Hexane (1.89)	338	6730	335	9040	352	2590
Toluene (2.38)	341	6460	339	8070	353	3120
DCM (8.93)	340	6030	334	10200	348	3370
2-propanol (17.9)	343	7820	341	11630	356	3620
THF (7.58)	344	5940	345	10000	359	4120
EtOAc (6.02)	342	7450	341	9640	354	3790
Dioxane (2.25)	343	5200	342	9390	356	3430
Acetone (20.7)	345	6100	341	12800	358	3430
Methanol (32.7)	342	3940	341	9410	353	4040
Pyridine (12.4)	347	5700	353	10890	362	4320
Acetonitrile (37.5)	342	8140	340	11980	355	3410
DMF (36.7)	346	7220	354	11430	362	3630
DMSO (46.7)	347	6170	359	13270	364	4460
Water (80.1)	336	8400	333	9330	340	2270

* The corresponding values for the 1,5-ICAN isomer are taken from ref [15] and listed here for comparison with those of the other ICAN isomers.

**Table 2:** The fluorescence emission maxima (λ_Em_) and the quantum yields (Φ_f_) determined in various solvents for the 1,5-ICAN, 1,4-ICAN, and 2,6-ICAN isomers.

	1,5-ICAN *		1,4-ICAN		2,6-ICAN	
Solvent (ε_r_)	λ_Em_ (nm)	Φ_f_	λ_Em_ (nm)	Φ_f_	λ_Em_ (nm)	Φ_f_
*n*-Hexane (1.89)	409	0.55	402	0.48	395	0.15
Toluene (2.38)	433	0.66	406	0.72	398	0.25
DCM (8.93)	461	0.88	411	0.74	398	0.29
2-propanol (17.9)	494	0.40	419	0.54	414	0.37
THF (7.58)	465	0.66	413	0.52	409	0.31
EtOAc (6.02)	464	0.45	411	0.64	406	0.24
Dioxane (2.25)	458	0.95	412	0.86	406	0.38
Acetone (20.7)	479	0.50	411	0.41	411	0.30
Methanol (32.7)	494	0.54	419	0.59	413	0.24
Pyridine (12.4)	490	0.28	423	0.05	420	0.09
Acetonitrile (37.5)	483	0.38	416	0.45	408	0.31
DMF (36.7)	491	0.48	422	0.69	419	0.53
DMSO (46.7)	497	0.74	425	0.70	420	0.51
Water (80.1)	513	0.04	434	0.63	419	0.21

* The corresponding values for the 1,5-ICAN isomer are taken from ref [15] and listed here for comparison with those of the other ICAN isomers.

**Table 3:** The Onsager-radius (a_o_), dipole moment difference between the excited and the ground state ((µ_E_ − µ_G_)_DFT_) calculated by DFT (in DMSO, the values in hexane are ~0.4 D lower), and the dipole moment differences in the excited and ground state ((µ_E_ − µ_G_)_LM_) determined by the Lippert–Mataga equation.

	a_o_ (pm)	(µ_E_ − µ_G_)_DFT_ (D)	(µ_E_ − µ_G_)_LM_ (D)
1,5-ICAN	305	4.8	4.9
1,4-ICAN	278	0.7	2.2
2,6-ICAN	388	3.5	3.6

**Table 4:** The fluorescence decay rate (k_F_), the radiative decay rate (k_r_), the non-radiative decay rate (k_nr_), and the radiative decay rate (k_SB_) calculated by the Strickler–Berg equation (Equation (5)) for the ICAN isomers.

	1,5-ICAN *				1,4-ICAN				2,6-ICAN			
Solvent	k_F_ × 10^−8^ (s^−1^)	k_r_ × 10^−8^ (s^−1^)	k_nr_ × 10^−8^ (s^−1^)	k_SB_ × 10^−8^ (s^−1^)	k_F_ × 10^−8^ (s^−1^)	k_r_ × 10^−8^ (s^−1^)	k_nr_ × 10^−8^ (s^−1^)	k_SB_ × 10^−8^ (s^−1^)	k_F_ × 10^−8^ (s^−1^)	k_r_× 10^−8^ (s^−1^)	k_nr_ × 10^−8^ (s^−1^)	k_SB_ × 10^−8^ (s^−1^)
*n*-Hexane	1.50	0.84	0.66	0.81	1.95	0.95	1.0	1.2	1.89	0.29	1.6	0.26
THF	0.93	0.62	0.31	0.74	1.42	0.74	0.68	1.1	0.91	0.28	0.63	0.29
MeOH	0.97	0.52	0.45	0.47	1.25	0.74	0.51	1.2	1.29	0.31	0.98	0.26
Acetonitrile	1.20	0.44	0.76	0.60	1.27	0.57	0.70	1.2	1.28	0.40	0.88	0.33
DMSO	0.62	0.46	0.16	0.71	0.71	0.50	0.21	1.7	0.71	0.36	0.35	0.37
Water	-	-	-	-	1.73	1.2	0.53	1.0	1.66	0.36	1.3	0.21

* The corresponding values for the 1,5-ICAN isomer were taken from ref [15] and listed here for comparison with those of the other ICAN isomers.

## Supplementary Materials

The Supplementary materials are available online, Figure S1: ^1^H-NMR spectrum of 2-amino-6-isocyanonaphthalene in chloroform, Figure S2: ^13^C-NMR spectrum of 2-amino-6-isocyanonaphthalene in chloroform, Figure S3: UV-vis spectra of 2-amino-6-isocyanonaphthalene, Figure S4: Normalized emission spectra of 2-amino-6-isocyanonaphthalene, Figure S5: Normalized excitation spectra of 2-amino-6-isocyanonaphthalene, Figure S6: Excitation spectra of 2-amino-6-isocyanonaphthalene, Figure S7: ^1^H-NMR spectrum of 1-amino-4-isocyanonaphthalene in chloroform, Figure S8: ^13^C-NMR spectrum of 1-amino-4-isocyanonaphthalene in chloroform, Figure S9: UV-vis spectra of 1-amino-4-isocyanonaphthalene, Figure S10: Normalized emission spectra of 1-amino-4-isocyanonaphthalene, Figure S11: Normalized excitation spectra of 1-amino-4-isocyanonaphthalene, Figure S12: Excitation spectra of 1-amino-4-isocyanonaphthalene, Table S1: Comparison of the results obtained by different functionals for the absorption wavelength calculations for the ICAN isomers in different solvents, Table S2: Comparison of the results obtained by different functionals for the emission wavelength calculations for the ICAN isomers in different solvents, Figure S13: Normalized calculated (**a)** and measured (**b**) emission spectra for the 1,4-ICAN, 1,5-ICAN and 2,6-ICAN isomers in DMSO, Figure S14: HOMO, LUMO, HOMO-1 (H-1) and LUMO+1 (L+1) molecular orbitals for the 1,4-ICAN (**a**), 1,5-ICAN (**b**) and 2,6-ICAN (**c**) isomer; Figure S15: Atomic indices for the calculation of the Mulliken charges presented in Tables S3–S5 for the 1,4-ICAN (**a**), 2,6-ICAN (**b**) and 1.5-ICAN (**c**) isomer, Table S3: Calculated atomic charges for the ground and excited states of the 1,4-ICAN isomer, Table S4: Calculated atomic charges for the ground and excited states of the 2,6-ICAN isomer, Table S5: Calculated atomic charges for the ground and excited states of the 1,5-ICAN isomer, Figure S16: Calculated electronic density differences calculated for the emission between the relaxed excited state and the corresponding vertical ground state in DMSO for the 1,4-ICAN (**a**) 2,6-ICAN (**b**) and 1,5-ICAN (**c**) isomers, Figure S17: Fluorescence decay of 1,4-ICAN in different solvents, Figure S18: Fluorescence decay of 2,6-ICAN in different solvents.
