# Supplementary material for: Effect of the Substitution Position on the Electronic and Solvatochromic Properties of Isocyanoaminonaphthalene (ICAN) Fluorophores
Source: Molecules. 2019 Jul 2;24(13):2434. doi: 10.3390/molecules24132434 (PMC6650821; doi:10.3390/molecules24132434)
Supplement: Supplementary file 1 [file molecules-24-02434-s001.pdf]

Supporting Information for

Effect of the substitution position on the electronic and solvatochromic properties of isocyanoaminonaphthalene (ICAN) fluorophores

Sándor Lajos Kovács[1], Miklós Nagy[1*], Péter Pál Fehér[2], Miklós Zsuga[1], Sándor Kéki[1]

[1]*Department of Applied Chemistry, University of Debrecen, Egyetem tér 1., H-4032 Debrecen, Hungary*

[2]*Institute of Organic Chemistry, Research Centre for Natural Sciences, Hungarian Academy of Sciences, Magyar tudósok körútja 2., H-1519 Budapest, Hungary*

* Corresponding author: miklos.nagy@science.unideb.hu, fax: +36 52 518662; *H-4032 Debrecen, HUNGARY*

Synthesis of 2-amino-6-isocyanonaphthalene

A 200 ml round-bottom flask was charged with 2,6-diaminonaphthalene (0.50 g, 3.16 mmol) dissolved in chloroform (80 ml) and with potassium hydroxide (7.00 g, 125 mmol) dissolved in water (5 ml) and vigorously stirred with a magnetic stirrer at 40 °C for a day in argon atmosphere. After cooling down, the organic phase was filtered, washed with water 3 times, dried on anhydrous magnesium sulfate and the solvent was removed on a rotary evaporator. The crude product was purified on a column filled with normal-phase silica gel, using dichloromethane as eluent. Yield: 0.550 g, 14% (pale yellow powder)

^{1}H NMR (360 MHz, Chloroform-*d*) δ 7.86 – 7.75 (m, 1H), 7.70 (d, *J* = 8.7 Hz, 1H), 7.63 (d, *J* = 8.7 Hz, 1H), 7.43 – 7.30 (m, 1H), 7.15 – 6.96 (m, 2H), 4.10 (s, 3H), 2.25 (s, 0H), 1.34 (s, 4H), 1.05 – 0.88 (m, 1H).

^{13}C NMR (91 MHz, Chloroform-*d*) δ 145.93 , 134.67 , 123.83 , 119.59 , 108.05 , 77.44 , 76.74 , 29.77 .

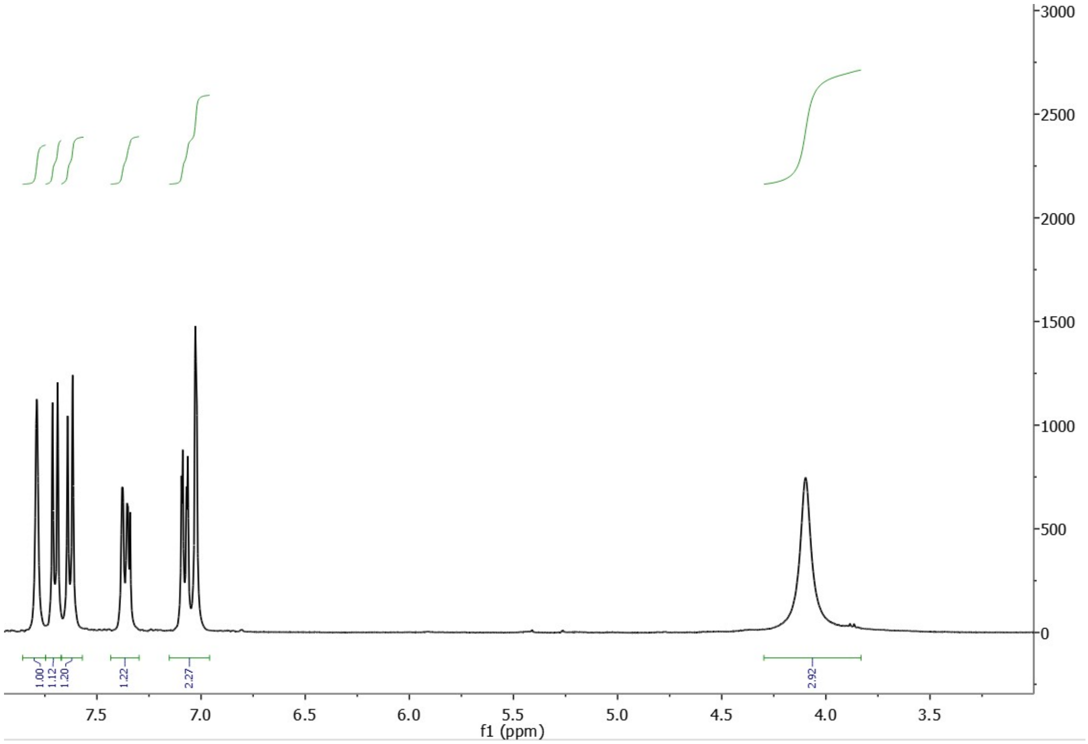

Fig. S1. ^{1}H-NMR spectrum of 2-amino-6-isocyanonaphthalene in chloroform.

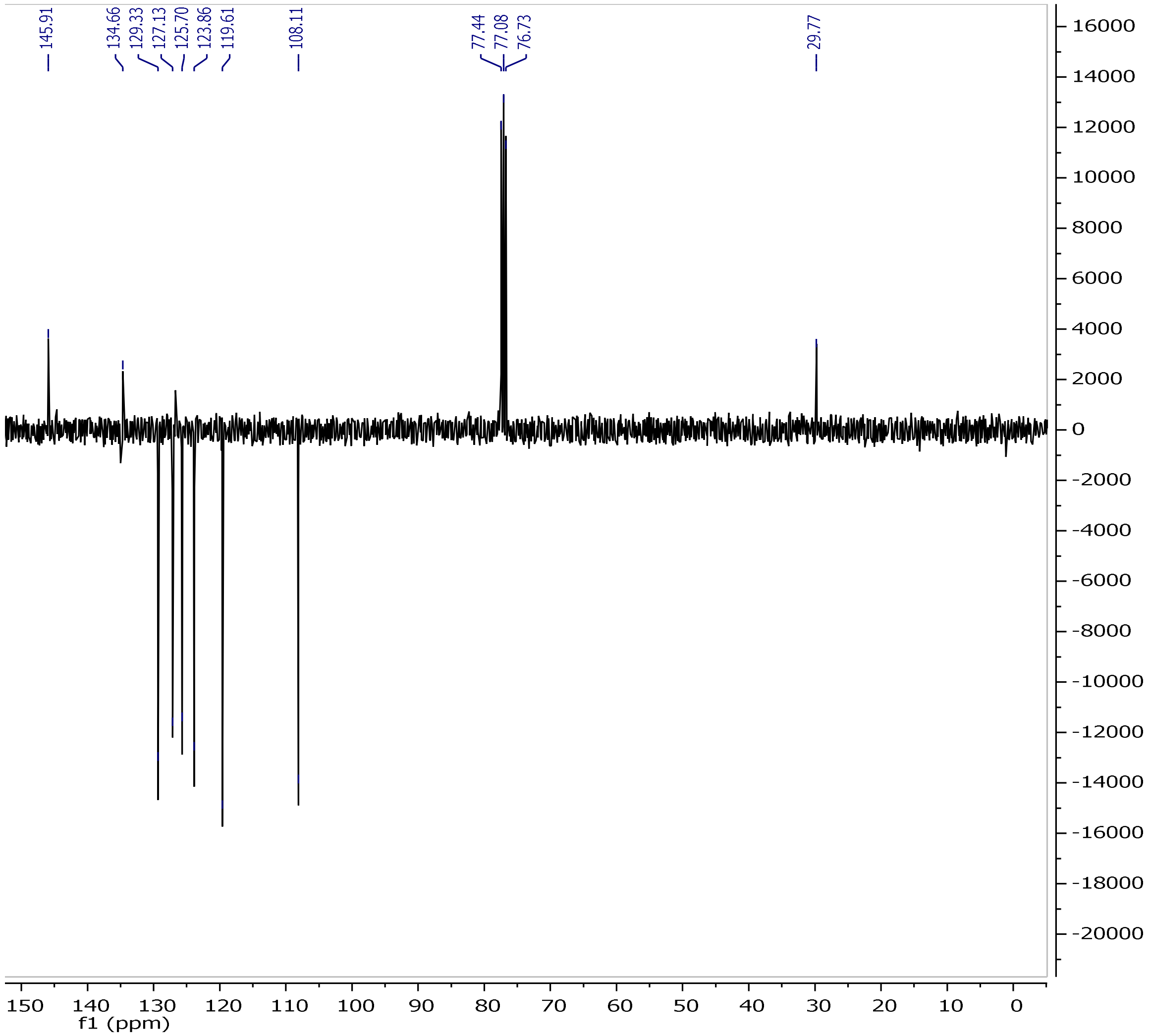

Fig. S2. ^{13}C-NMR spectrum of 2-amino-6-isocyanonaphthalene in chloroform.

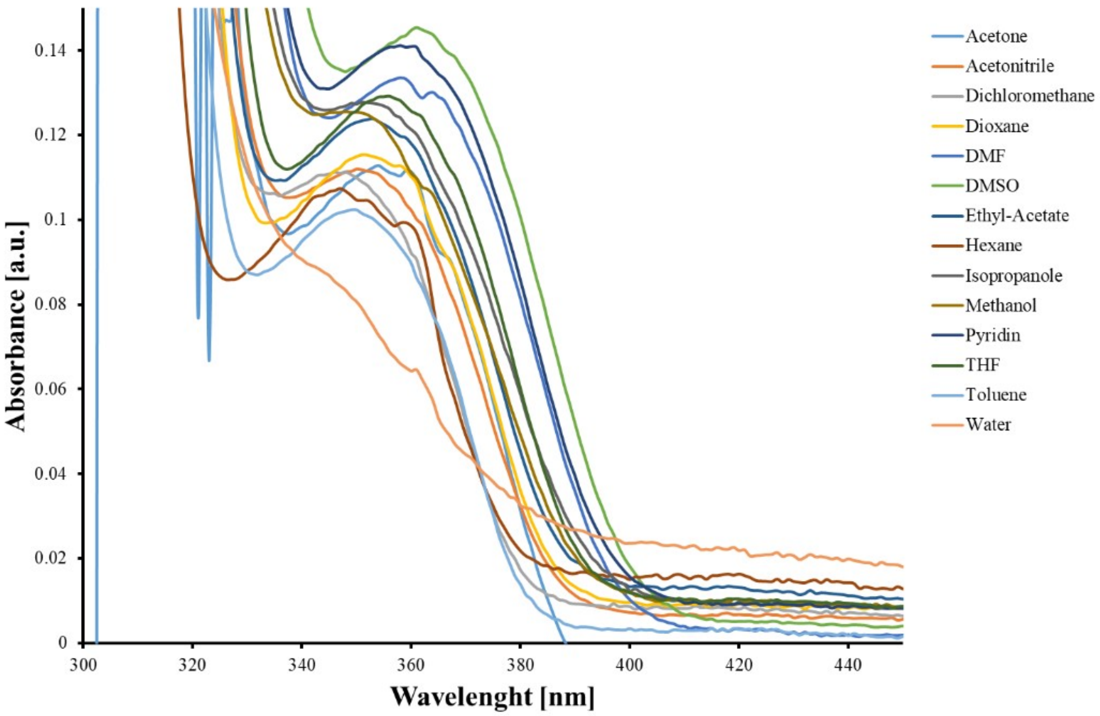

Fig. S3. UV-vis spectra of 2-amino-6-isocyanonaphthalene 20°C, V = 3 cm^3, c = 2.4x10^{-5} M

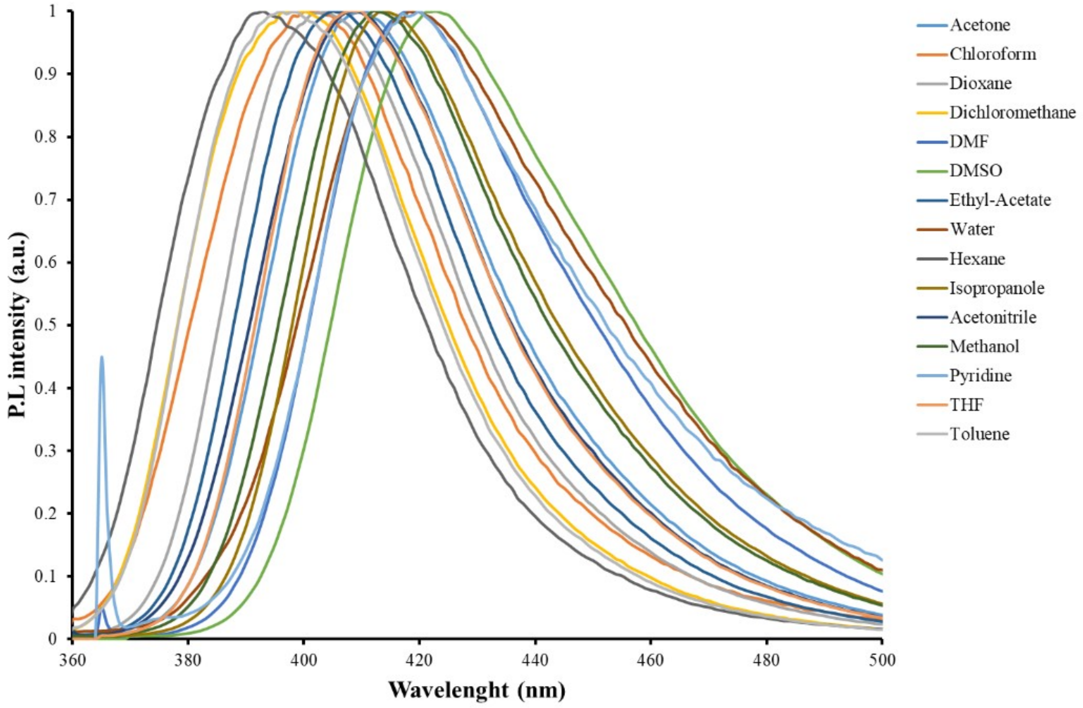

Fig. S4. Normalized emission spectra of 2-amino-6-isocyanonaphthalene 20°C, V = 3 cm^3, c = 4.12x 10^{-6} M

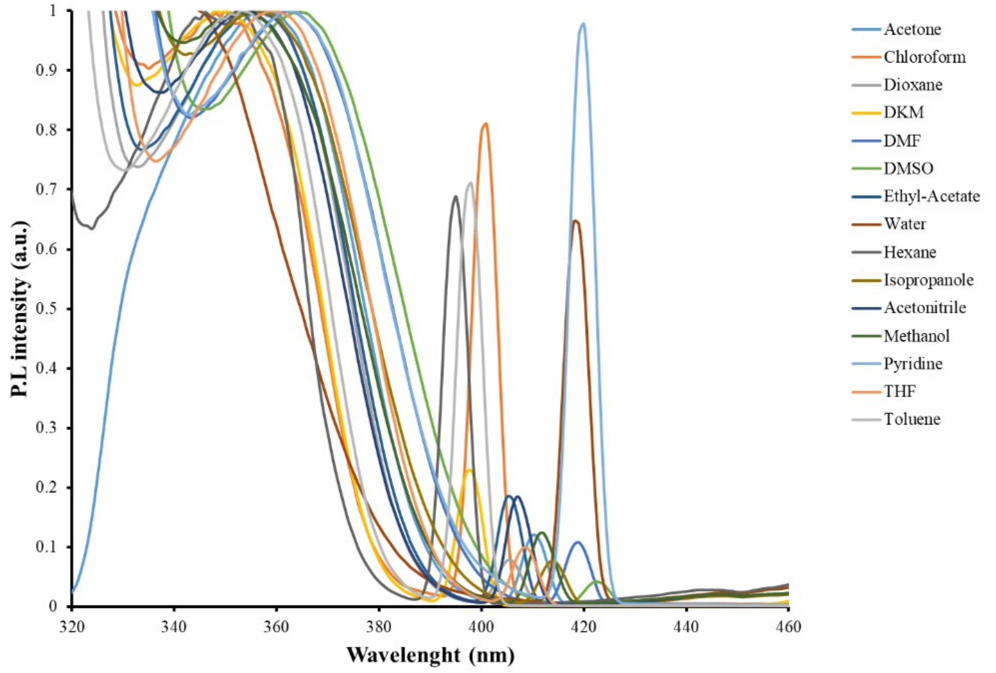

Fig. S5. Normalized excitation spectra of 2-amino-6-isocyanonaphthalene 20°C, V = 3 cm^3, c = 4.12· 10^{-6} M

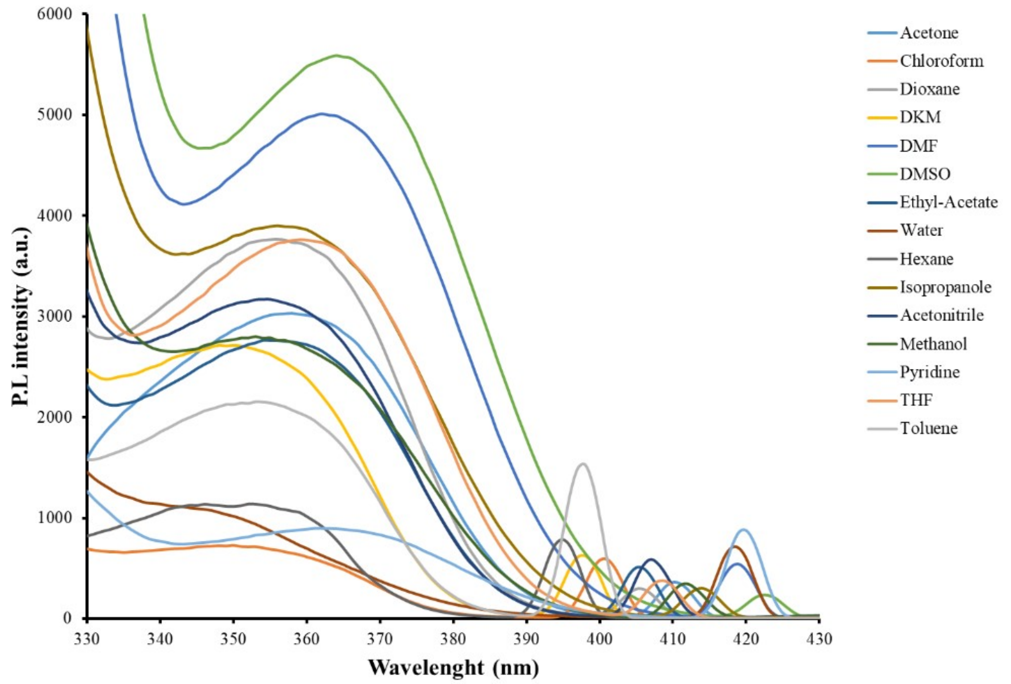

Fig. S6. Excitation spectra of 2-amino-6-isocyanonaphthalene 20°C, V = 3 cm^3, c = 4.12· 10^{-6} M

Synthesis of 1-amino-4-isocyanonaphthalene

A 200 ml round-bottom flask was charged with 1,4-diaminonaphthalene hydrochloride salt (2,00 g, 12,8 mmol) dissolved in chloroform (20 ml) and with potassium hydroxide (10.0 g, 536 mmol) dissolved in water (5 ml) and vigorously stirred with a magnetic stirrer at 40 °C for 6 hours in argon atmosphere. After cooling down, the organic phase was filtered, dried on anhydrous magnesium sulfate and the solvent was removed on a rotary evaporator. The crude product was purified on a column filled with normal-phase silica gel, using dichloromethane as eluent. Yield: 0.50 g, 25% (yellow powder)

^{1}H NMR (400 MHz, Chloroform-*d*) δ 8.16 (d, *J* = 8.4 Hz, 1H), 7.84 (d, *J* = 8.4 Hz, 1H), 7.73 – 7.64 (m, 1H), 7.58 (t, *J* = 8.2 Hz, 1H), 7.44 (d, *J* = 8.0 Hz, 1H), 7.28 (s, 0H), 6.68 (d, *J* = 8.0 Hz, 1H), 5.32 (s, 0H), 4.47 (s, 3H).

^{13}C NMR (101 MHz, Chloroform-*d*) δ 164.68 , 143.85 , 129.03 , 127.91 , 126.10 , 125.75 , 107.65 , 77.35 , 76.87 (d, *J* = 31.9 Hz), 29.70 .

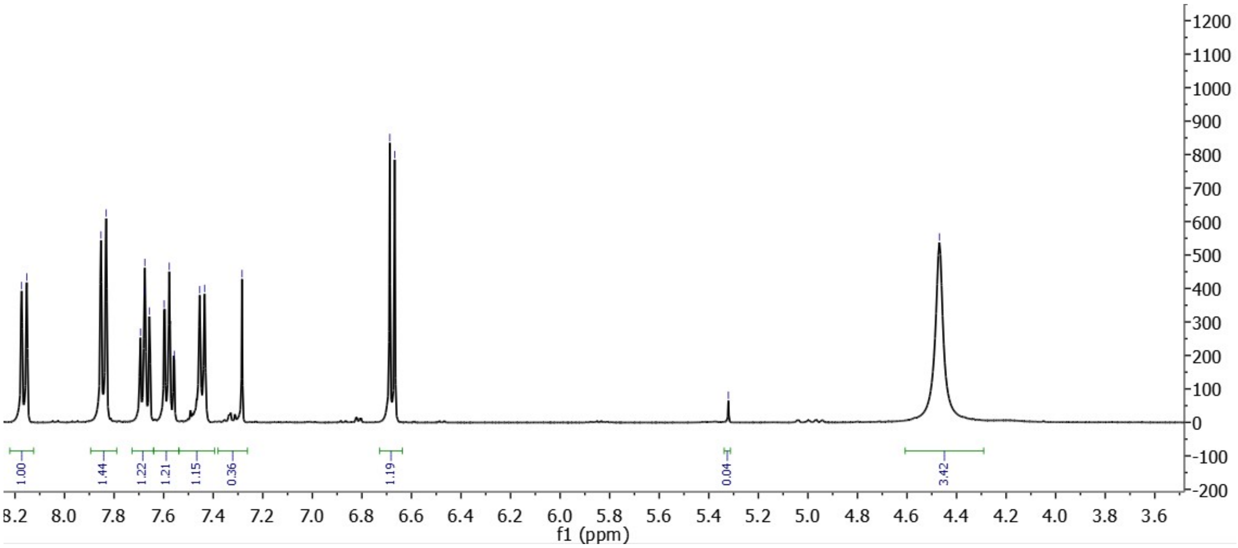

Fig. S7. ^{1}H-NMR spectrum of 1-amino-4-isocyanonaphthalene in chloroform

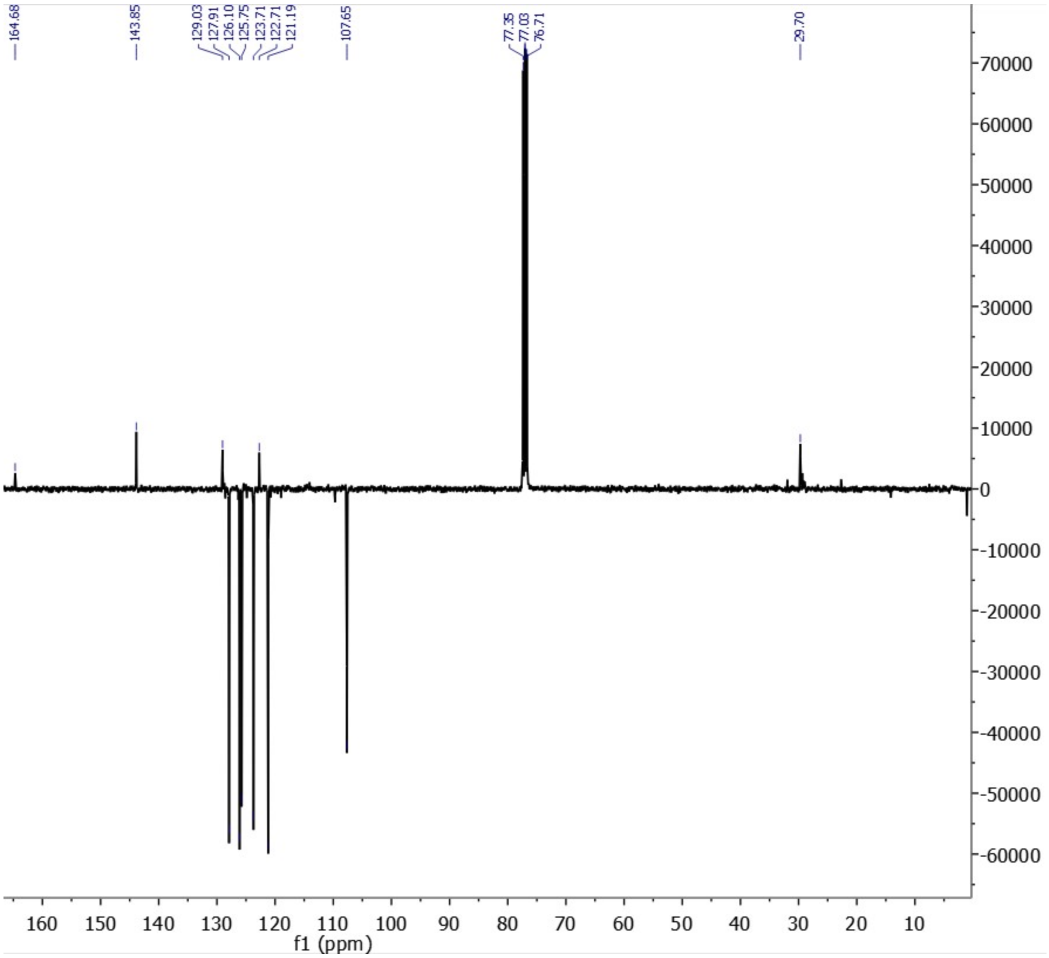

Fig. S8. ^{13}C-NMR spectrum of 1-amino-4-isocyanonaphthalene in chloroform

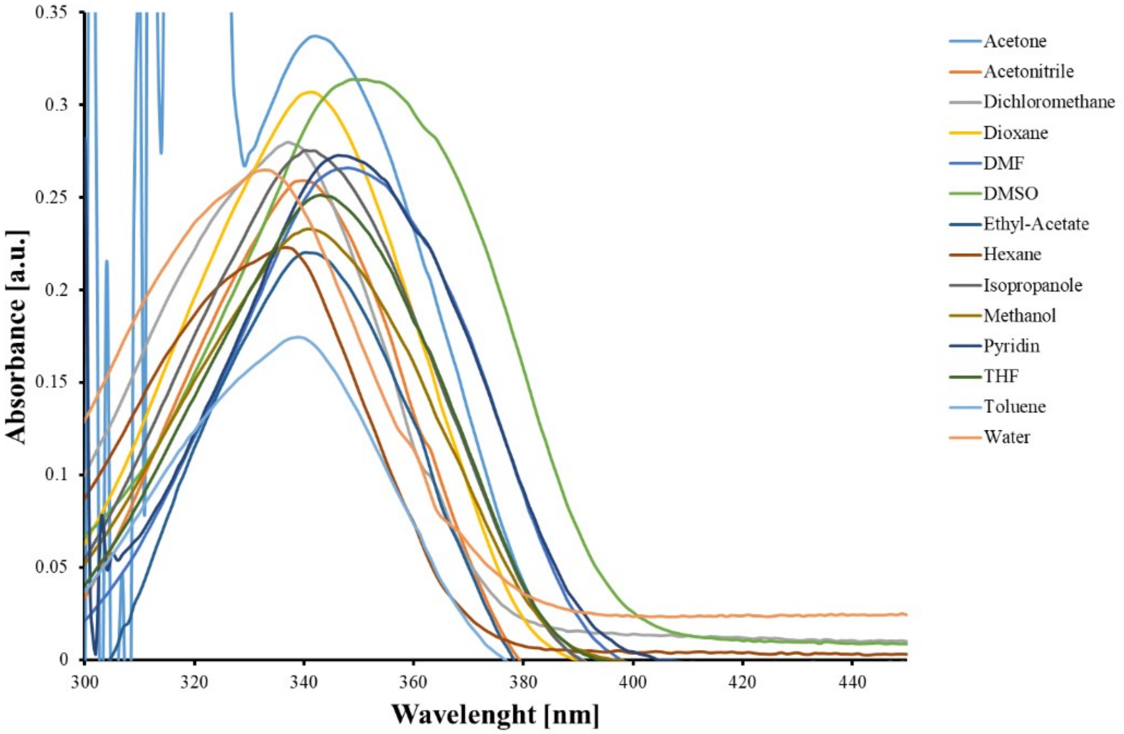

Fig. S9. UV-vis spectra of 1-amino-4-isocyanonaphthalene 20°C, V = 3 cm^3, c = $2.4 \cdot 10^{-5}$ M

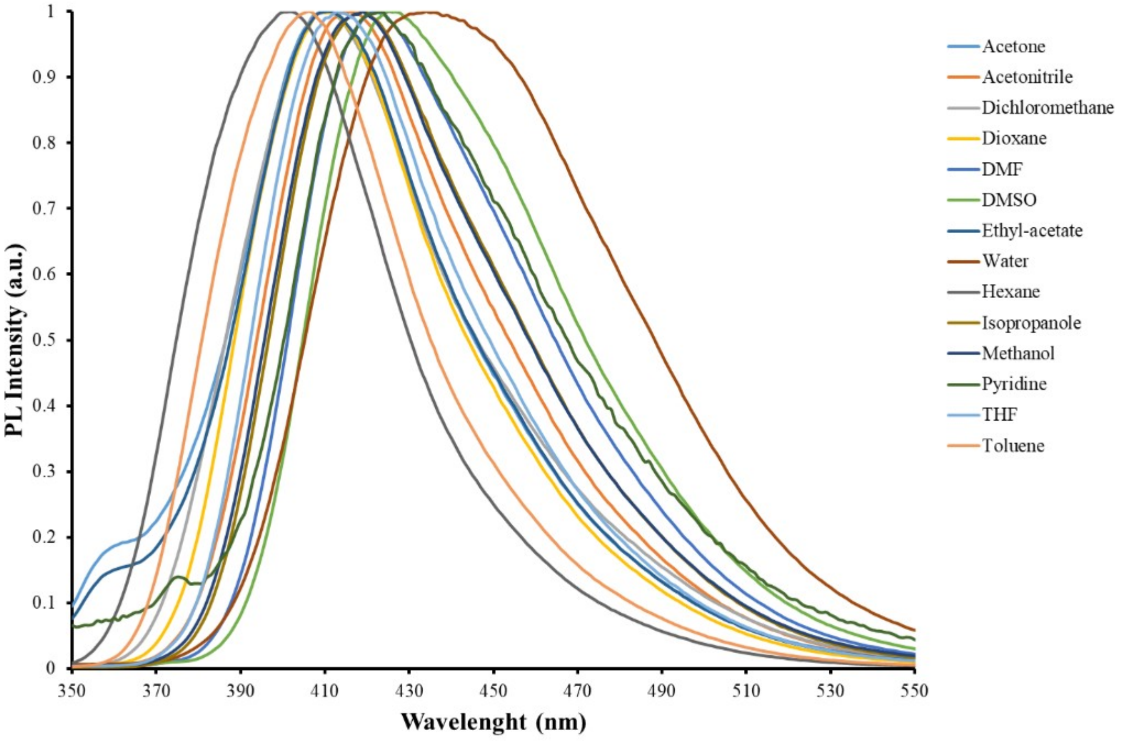

Fig. S10. Normalized emission spectra of 1-amino-4-isocyanonaphthalene 20°C, V = 3 cm^3, c = $4.12 \cdot 10^{-6}$ M

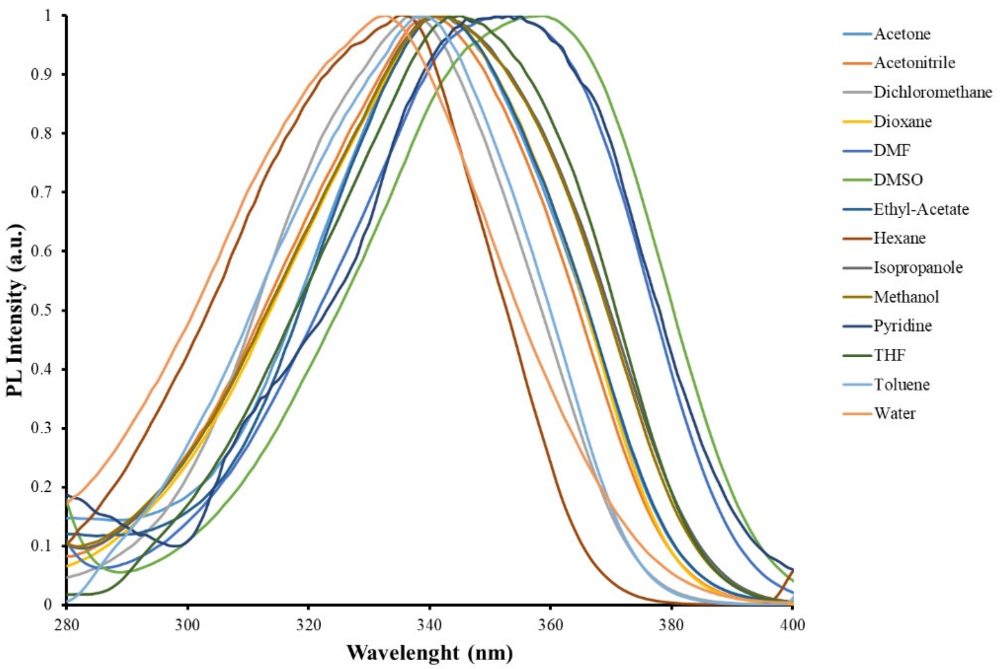

Fig. S11. Normalized excitation spectra of 1-amino-4-isocyanonaphthalene 20°C, V = 3 cm^3, c = 4.12· 10^{-6} M

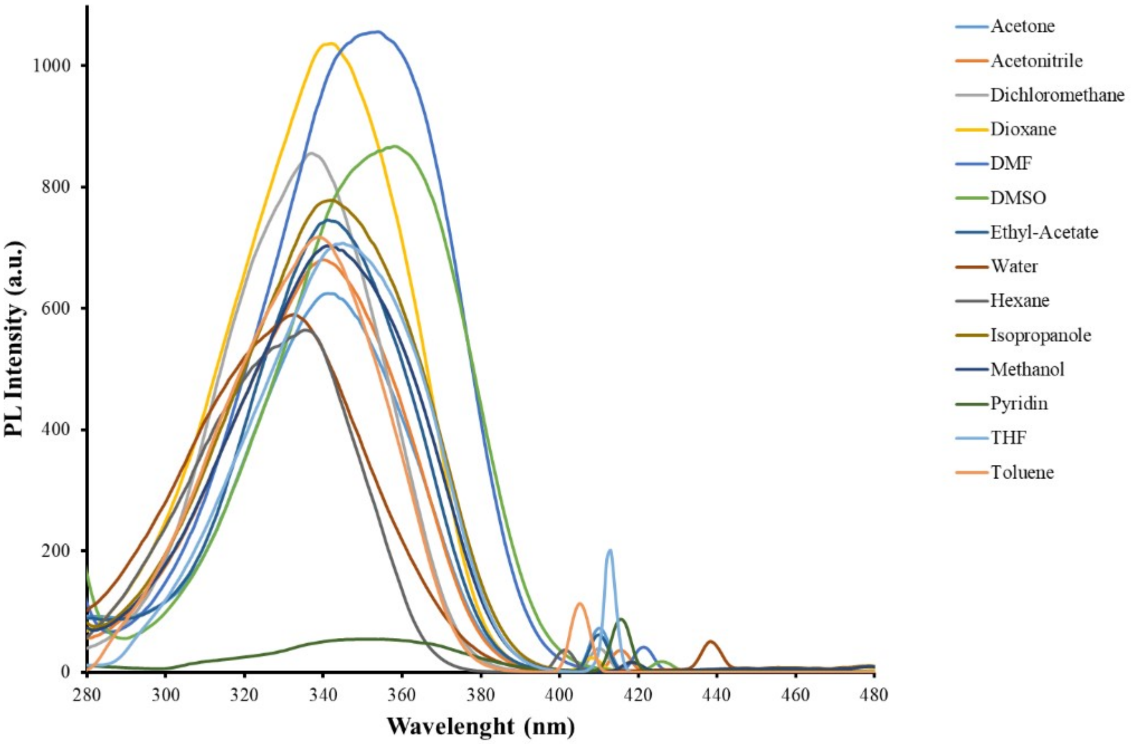

Fig. S12. Excitation spectra of 1-amino-4-isocyanonaphthalene 20°C, V = 3 cm^3, c = 4.12· 10^{-6} M

Table S1. Comparison of the results obtained by different functionals for the absorption wavelength calculations for the ICAN isomers in different solvents. The last column indicates the number of isomers for which the calculated data was inside 30 nm of the measured one. The higher this value is, the higher is the precision of the given functional.

	**ABSORPTION**	**1,5-ICAN**		**1,4-ICAN**		**2,6-ICAN**		
	**method**	**λ**	**Δλ**	**λ**	**Δλ**	**λ**	**Δλ**	**nº inside 30 nm (~8.5%)**
n-hexane	CAM-B3LYP (cLR on M06 geom.)							
n-hexane	CAM-opt (cLR)	319	-19	303	-32	303	-49	**1**
n-hexane	CAM-opt (eq)	319	-19	306	-29	304	-48	**2**
n-hexane	M06 (cLR)	363	25	335	0	332	-20	**3**
**n-hexane**	**EXP**	**338**	**0**	**335**	**0**	**352**	**0**	**-**
DMSO	CAM-B3LYP (cLR on M06 geom.)	331	-16					**1**
DMSO	CAM-opt (cLR)	326	-21	306	-53	305	-59	**1**
DMSO	CAM-opt (eq)	323	-24	310	-49	305	-59	**1**
DMSO	M06 (cLR)	375	28	337	-22	336	-28	**3**
**DMSO**	**EXP**	**347**	**0**	**359**	**0**	**364**	**0**	**-**
water	CAM-B3LYP (cLR on M06 geom.)	316	-20					**1**
water	CAM-opt (cLR)	311	-25					**1**
water	CAM-opt (eq)	310	-26					**1**
water	M06 (eq)	351	15					**1**
water	M06 (cLR)	355	19					**1**
**water**	**EXP**	**336**	0					**-**

Table S2. Comparison of the results obtained by different functionals for the emission wavelength calculations for the ICAN isomers in different solvents. The last column indicates the number of isomers for which the calculated data was inside 30 nm of the measured one. The higher this value is, the higher is the precision of the given functional.

	**EMISSION**	**1,5-ICAN**		**1,4-ICAN**		**2,6-ICAN**		
	**method**	**λ**	**Δλ**	**λ**	**Δλ**	**λ**	**Δλ**	**nº inside 30 nm (~7.5%)**
n-hexane	CAM-B3LYP (cLR on M06 geom.)	391	-18	361	-41	346	-49	**1**
n-hexane	CAM-opt (cLR)	392	-17	362	-40	346	-49	**1**
n-hexane	CAM-opt (eq)	392	-17	368	-34	348	-47	**1**
n-hexane	M06 (cLR)	440	31	392	-10	375	-20	**2**
**n-hexane**	**EXP**	**409**	**0**	**402**	**0**	**395**	**0**	**-**
DMSO	CAM-B3LYP (cLR on M06 geom.)	429	-68	367	-58	359	-61	**0**
DMSO	CAM-opt (cLR)	427	-70	368	-57	361	-59	**0**
DMSO	CAM-opt (eq)	416	-81	386	-39	365	-55	**0**
DMSO	M06 (cLR)	495	-2	399	-26	392	-28	**3**
**DMSO**	**EXP**	**497**	**0**	**425**	**0**	**420**	**0**	**-**
water	CAM-opt (cLR)	460	-53					**0**
water	CAM-opt (eq)	434	-79					**0**
water	M06 (opt)	484	-29					**1**
water	M06 (cLR)	537	24					**1**
**water**	**EXP**	**513**	0					**-**

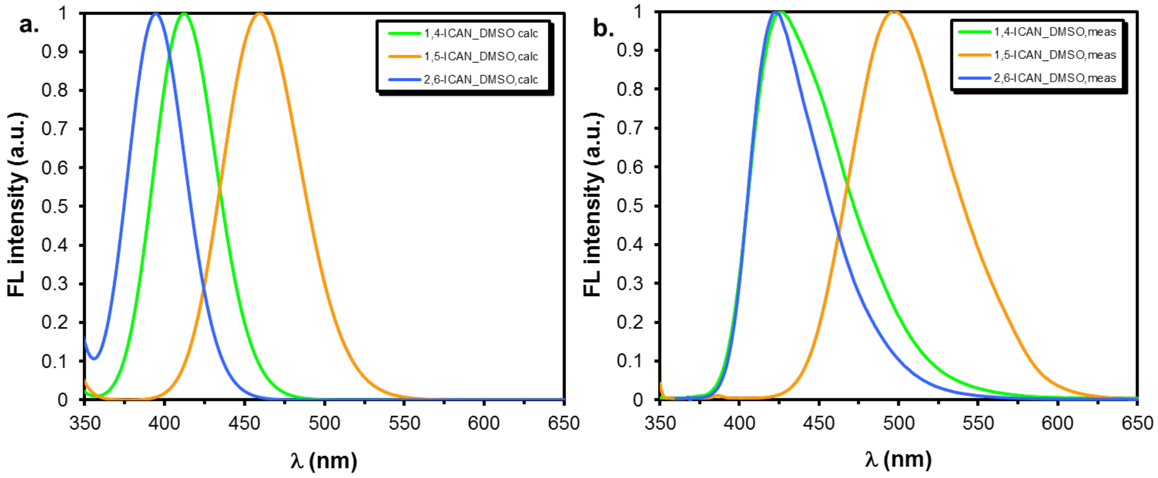

Figure S13. Normalized calculated (**a**) and measured (**b**) emission spectra for the 1,4-ICAN, 1,5-ICAN and 2,6-ICAN isomers in DMSO. The measured fluorescence emission spectrum for the 1,5-ICAN are taken from Ref. [15].

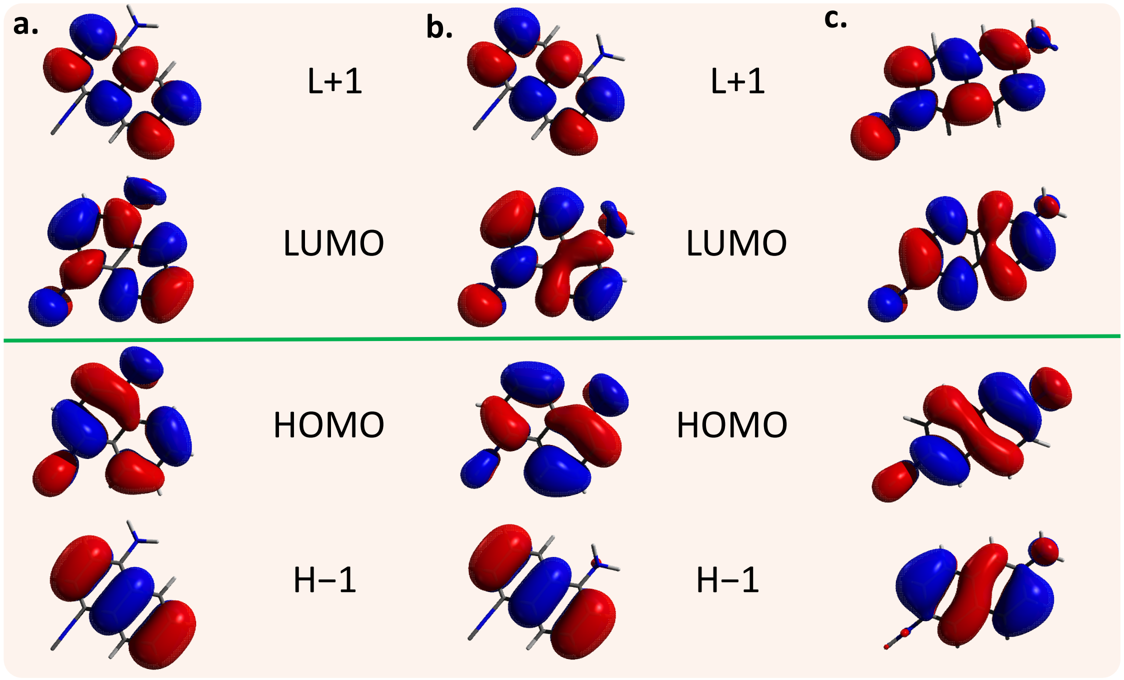

Figure S14. HOMO, LUMO, HOMO-1 (H-1) and LUMO+1 (L+1) molecular orbitals for the 1,4-ICAN (**a**), 1,5-ICAN (**b**) and 2,6-ICAN (**c**) isomer.

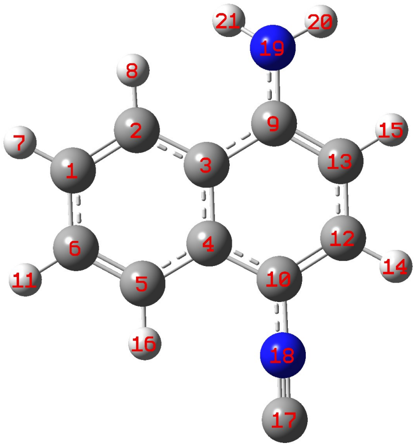

a

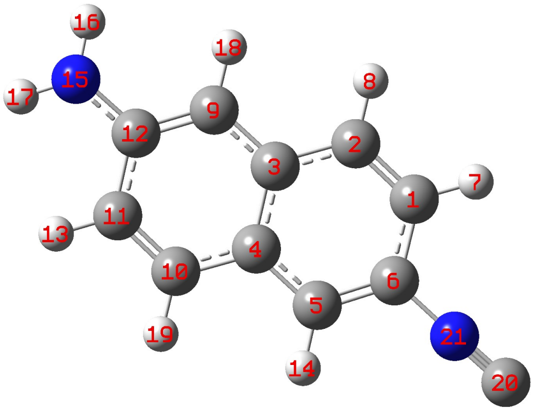

b

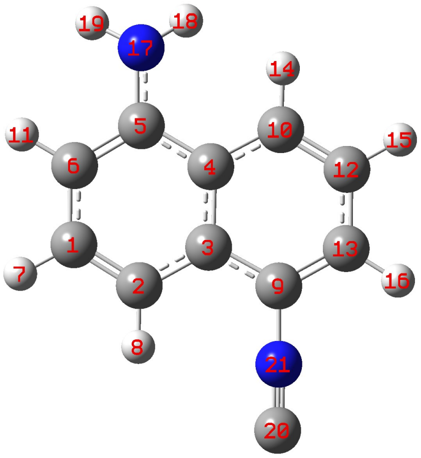

c

Fig. S15. Atomic indices for the calculation of the Mulliken charges presented in Tables S3-S5 for the 1,4-ICAN (**a**), 2,6-ICAN (**b**) and 1.5-ICAN (**c**) isomer.

Table S3. Calculated atomic charges for the ground and excited states of the 1,4-ICAN isomer.

		Ground State	S1 (vertical)		S1 (relaxed)		
		Mulliken	Mulliken	ΔQ	Mulliken	ΔQ (GS)	ΔQ (S1-vert.)
1	C	-0.1503	-0.1671	-0.0167	-0.1656	-0.0153	0.0015
2	C	-0.0899	-0.1662	-0.0763	-0.1602	-0.0703	0.0060
3	C	-0.1707	-0.1374	0.0333	-0.2337	-0.0630	-0.0963
4	C	-0.3212	-0.3241	-0.0029	-0.2707	0.0506	0.0535
5	C	-0.0458	-0.1019	-0.0561	-0.0915	-0.0457	0.0104
6	C	-0.1579	-0.1986	-0.0407	-0.2019	-0.0439	-0.0033
7	H	0.1573	0.1502	-0.0071	0.1513	-0.0060	0.0010
8	H	0.1719	0.1678	-0.0041	0.1554	-0.0165	-0.0124
9	C	0.1300	0.1232	-0.0068	0.3034	0.1734	0.1802
10	C	0.1676	0.2236	0.0560	0.1680	0.0004	-0.0557
11	H	0.1605	0.1512	-0.0093	0.1525	-0.0081	0.0013
12	C	-0.1604	-0.2156	-0.0552	-0.1946	-0.0342	0.0210
13	C	-0.1502	-0.0943	0.0559	-0.1742	-0.0240	-0.0799
14	H	0.1821	0.1895	0.0074	0.1919	0.0098	0.0024
15	H	0.1722	0.1905	0.0183	0.1914	0.0193	0.0009
16	H	0.1741	0.1694	-0.0048	0.1691	-0.0051	-0.0003
17	C	-0.1059	-0.1020	0.0039	-0.1109	-0.0050	-0.0089
18	N	-0.1133	-0.1020	0.0113	-0.1002	0.0131	0.0018
19	N	-0.3778	-0.3113	0.0665	-0.3731	0.0047	-0.0618
20	H	0.2659	0.2797	0.0138	0.2977	0.0318	0.0180
21	H	0.2617	0.2754	0.0137	0.2960	0.0342	0.0205

hydrogens summed into heavy atoms

		Mulliken	Mulliken	ΔQ	Mulliken	ΔQ (GS)	ΔQ (S1-vert.)
1	C	0.0070	-0.0168	-0.0238	-0.0143	-0.0213	0.0025
2	C	0.0820	0.0016	-0.0804	-0.0048	-0.0868	-0.0064
3	C	-0.1707	-0.1374	0.0333	-0.2337	-0.0630	-0.0963
4	C	-0.3212	-0.3241	-0.0029	-0.2707	0.0506	0.0535
5	C	0.1283	0.0674	-0.0609	0.0776	-0.0508	0.0101
6	C	0.0026	-0.0474	-0.0500	-0.0494	-0.0520	-0.0020
9	C	0.1300	0.1232	-0.0068	0.3034	0.1734	0.1802
10	C	0.1676	0.2236	0.0560	0.1680	0.0004	-0.0557
12	C	0.0217	-0.0261	-0.0478	-0.0027	-0.0244	0.0234
13	C	0.0220	0.0962	0.0742	0.0172	-0.0048	-0.0789
17	C	-0.1059	-0.1020	0.0039	-0.1109	-0.0050	-0.0089
18	N	-0.1133	-0.1020	0.0113	-0.1002	0.0131	0.0018
19	N	0.1498	0.2438	0.0940	0.2206	0.0708	-0.0232

Table S4. . Calculated atomic charges for the ground and excited states of the 2,6-ICAN isomer.

		Ground State	S1 (vertical)		S1 (relaxed)		
		Mulliken	Mulliken	ΔQ	Mulliken	ΔQ (GS)	ΔQ (S1-vert.)
1	C	-0.1950	-0.2266	-0.0316	-0.2131	-0.0181	0.0135
2	C	-0.0902	-0.1302	-0.0400	-0.1253	-0.0351	0.0049
3	C	-0.1820	-0.1932	-0.0112	-0.2265	-0.0446	-0.0334
4	C	-0.1871	-0.1599	0.0272	-0.1425	0.0446	0.0174
5	C	-0.0787	-0.1857	-0.1070	-0.2038	-0.1252	-0.0182
6	C	0.0593	0.0873	0.0280	0.0938	0.0344	0.0065
7	H	0.1919	0.1858	-0.0060	0.1879	-0.0040	0.0021
8	H	0.1623	0.1576	-0.0047	0.1602	-0.0021	0.0026
9	C	-0.0924	-0.0443	0.0481	-0.0987	-0.0064	-0.0545
10	C	-0.1035	-0.1239	-0.0204	-0.1410	-0.0375	-0.0171
11	C	-0.1748	-0.2235	-0.0487	-0.2184	-0.0435	0.0052
12	C	0.0493	0.0737	0.0244	0.1844	0.1350	0.1106
13	H	0.1862	0.1856	-0.0006	0.1877	0.0015	0.0021
14	H	0.1952	0.1897	-0.0055	0.1865	-0.0087	-0.0032
15	N	-0.4003	-0.3050	0.0952	-0.3585	0.0418	-0.0534
16	H	0.2608	0.2821	0.0213	0.3018	0.0410	0.0197
17	H	0.2618	0.2878	0.0260	0.3001	0.0383	0.0124
18	H	0.1802	0.2041	0.0239	0.2016	0.0215	-0.0025
19	H	0.1616	0.1566	-0.0050	0.1554	-0.0062	-0.0013
20	C	-0.1076	-0.1239	-0.0163	-0.1354	-0.0278	-0.0115
21	N	-0.0970	-0.0942	0.0029	-0.0962	0.0008	-0.0021

hydrogens summed into heavy atoms

		Mulliken	Mulliken	ΔQ	Mulliken	ΔQ (GS)	ΔQ (S1-vert.)
1	C	-0.0032	-0.0408	-0.0376	-0.0252	-0.0221	0.0156
2	C	0.0721	0.0274	-0.0447	0.0350	-0.0372	0.0075
3	C	-0.1820	-0.1932	-0.0112	-0.2265	-0.0446	-0.0334
4	C	-0.1871	-0.1599	0.0272	-0.1425	0.0446	0.0174
5	C	0.1165	0.0040	-0.1125	-0.0173	-0.1338	-0.0213
6	C	0.0593	0.0873	0.0280	0.0938	0.0344	0.0065
9	C	0.0878	0.1598	0.0720	0.1029	0.0151	-0.0569
10	C	0.0582	0.0327	-0.0254	0.0144	-0.0437	-0.0183
11	C	0.0113	-0.0379	-0.0492	-0.0307	-0.0420	0.0073
12	C	0.0493	0.0737	0.0244	0.1844	0.1350	0.1106
15	N	0.1223	0.2648	0.1425	0.2435	0.1212	-0.0213
20	C	-0.1076	-0.1239	-0.0163	-0.1354	-0.0278	-0.0115
21	N	-0.0970	-0.0942	0.0029	-0.0962	0.0008	-0.0021

Table S5. . Calculated atomic charges for the ground and excited states of the 1,5-ICAN isomer.

		Ground State	Hirshfeld		S1 (vertical)		Hirshfeld					S1 (relaxed)	
		Mulliken	Q-H	Q-CM5	Mulliken	ΔQ	Q-H	Q-CM5	ΔQ (H)	ΔQ (CM5)	Mulliken	ΔQ (GS)	ΔQ (S1-vert.)
1	C	-0.1845	-0.0338	-0.0891	-0.1929	-0.0084	-0.0260	-0.0812	0.0079	0.0079	-0.1804	0.0041	0.0125
2	C	-0.0443	-0.0729	-0.1236	0.0645	0.1087	0.0119	-0.0387	0.0849	0.0849	0.0237	0.0680	-0.0407
3	C	-0.3143	-0.0047	-0.0032	-0.3531	-0.0389	-0.0123	-0.0108	-0.0075	-0.0075	-0.3311	-0.0168	0.0220
4	C	-0.1770	-0.0168	-0.0167	-0.1255	0.0515	-0.0108	-0.0107	0.0060	0.0060	-0.1852	-0.0082	-0.0597
5	C	0.0953	0.0545	0.1085	0.1116	0.0163	0.0916	0.1457	0.0371	0.0372	0.2630	0.1676	0.1514
6	C	-0.1328	-0.0720	-0.1201	-0.0637	0.0691	-0.0125	-0.0606	0.0595	0.0595	-0.1362	-0.0034	-0.0725
7	H	0.1539	0.0483	0.1044	0.1682	0.0143	0.0570	0.1131	0.0087	0.0087	0.1684	0.0145	0.0002
8	H	0.1736	0.0319	0.0938	0.1995	0.0259	0.0541	0.1159	0.0221	0.0221	0.1938	0.0202	-0.0057
9	C	0.1510	0.0488	0.1113	0.1337	-0.0173	0.0002	0.0627	-0.0486	-0.0486	0.1470	-0.0040	0.0133
10	C	-0.0590	-0.0294	-0.0811	-0.1666	-0.1076	-0.1003	-0.1521	-0.0710	-0.0710	-0.1411	-0.0821	0.0255
11	H	0.1659	0.0432	0.1025	0.1913	0.0254	0.0635	0.1228	0.0203	0.0203	0.1936	0.0277	0.0023
12	C	-0.1602	-0.0366	-0.0921	-0.1593	0.0010	-0.0521	-0.1076	-0.0155	-0.0155	-0.1664	-0.0061	-0.0071
13	C	-0.1308	-0.0275	-0.0740	-0.2330	-0.1022	-0.0772	-0.1236	-0.0497	-0.0497	-0.2435	-0.1127	-0.0105
14	H	0.1810	0.0484	0.1094	0.1726	-0.0084	0.0251	0.0861	-0.0233	-0.0233	0.1552	-0.0258	-0.0174
15	H	0.1590	0.0528	0.1090	0.1496	-0.0094	0.0421	0.0983	-0.0107	-0.0107	0.1502	-0.0089	0.0005
16	H	0.1874	0.0577	0.1176	0.1731	-0.0143	0.0372	0.0971	-0.0205	-0.0205	0.1736	-0.0138	0.0005
17	N	-0.3905	-0.1681	-0.6250	-0.2949	0.0956	-0.0769	-0.5338	0.0912	0.0912	-0.3554	0.0351	-0.0605
18	H	0.2577	0.1262	0.3176	0.2834	0.0257	0.1527	0.3441	0.0265	0.0265	0.3043	0.0465	0.0208
19	H	0.2591	0.1337	0.3244	0.2837	0.0246	0.1609	0.3515	0.0272	0.0272	0.3055	0.0464	0.0218
20	C	-0.0808	-0.1336	-0.0197	-0.2086	-0.1278	-0.2480	-0.1341	-0.1144	-0.1144	-0.2123	-0.1315	-0.0037
21	N	-0.1099	-0.0502	-0.2538	-0.1337	-0.0239	-0.0803	-0.2840	-0.0301	-0.0302	-0.1267	-0.0169	0.0070

hydrogens summed into heavy atoms

		Mulliken	Q-H	Q-CM5	Mulliken	ΔQ	Q-H	Q-CM5	**ΔQ (H)**	**ΔQ (CM5)**	Mulliken	**ΔQ (GS)**	**ΔQ (S1-vert.)**
1	C	-0.0306	0.0145	0.0153	-0.0247	0.0059	0.0311	0.0319	0.0166	0.0166	-0.0119	0.0186	0.0127
2	C	0.1294	-0.0410	-0.0298	0.2640	0.1346	0.0660	0.0772	0.1070	0.1070	0.2176	0.0882	-0.0464
3	C	-0.3143	-0.0047	-0.0032	-0.3531	-0.0389	-0.0123	-0.0108	-0.0075	-0.0075	-0.3311	-0.0168	0.0220
4	C	-0.1770	-0.0168	-0.0167	-0.1255	0.0515	-0.0108	-0.0107	0.0060	0.0060	-0.1852	-0.0082	-0.0597
5	C	0.0953	0.0545	0.1085	0.1116	0.0163	0.0916	0.1457	0.0371	0.0372	0.2630	0.1676	0.1514
6	C	0.0330	-0.0287	-0.0176	0.1276	0.0945	0.0510	0.0621	0.0798	0.0798	0.0574	0.0243	-0.0702
9	C	0.1510	0.0488	0.1113	0.1337	-0.0173	0.0002	0.0627	-0.0486	-0.0486	0.1470	-0.0040	0.0133
10	C	0.1220	0.0191	0.0283	0.0060	-0.1160	-0.0752	-0.0660	-0.0943	-0.0943	0.0142	-0.1079	0.0082
12	C	-0.0012	0.0162	0.0169	-0.0097	-0.0085	-0.0100	-0.0093	-0.0262	-0.0262	-0.0162	-0.0150	-0.0066
13	C	0.0566	0.0302	0.0437	-0.0599	-0.1165	-0.0399	-0.0265	-0.0701	-0.0701	-0.0699	-0.1265	-0.0100
17	N	0.1263	0.0919	0.0170	0.2723	0.1460	0.2368	0.1619	0.1449	0.1449	0.2543	0.1280	-0.0180
20	C	-0.0808	-0.1336	-0.0197	-0.2086	-0.1278	-0.2480	-0.1341	-0.1144	-0.1144	-0.2123	-0.1315	-0.0037
21	N	-0.1099	-0.0502	-0.2538	-0.1337	-0.0239	-0.0803	-0.2840	-0.0301	-0.0302	-0.1267	-0.0169	0.0070

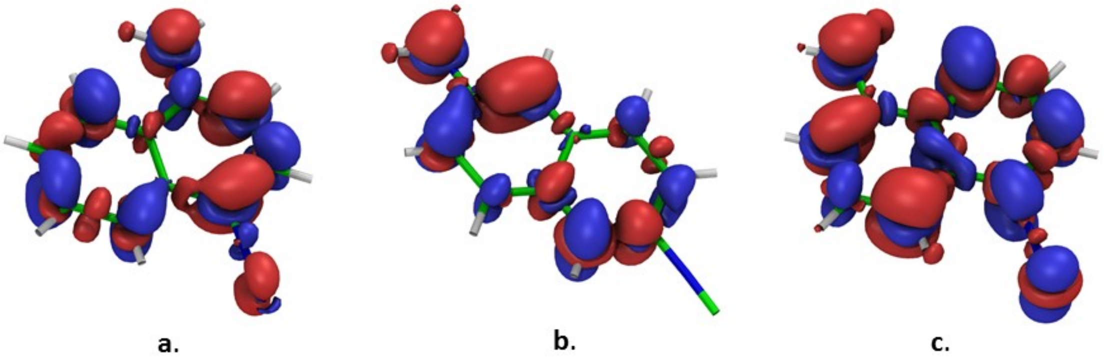

Figure S16. Calculated electronic density differences calculated for the emission between the relaxed excited state and the corresponding vertical ground state in DMSO for the 1,4-ICAN (**a**) 2,6-ICAN (**b**) and 1,5-ICAN (**c**) isomers.

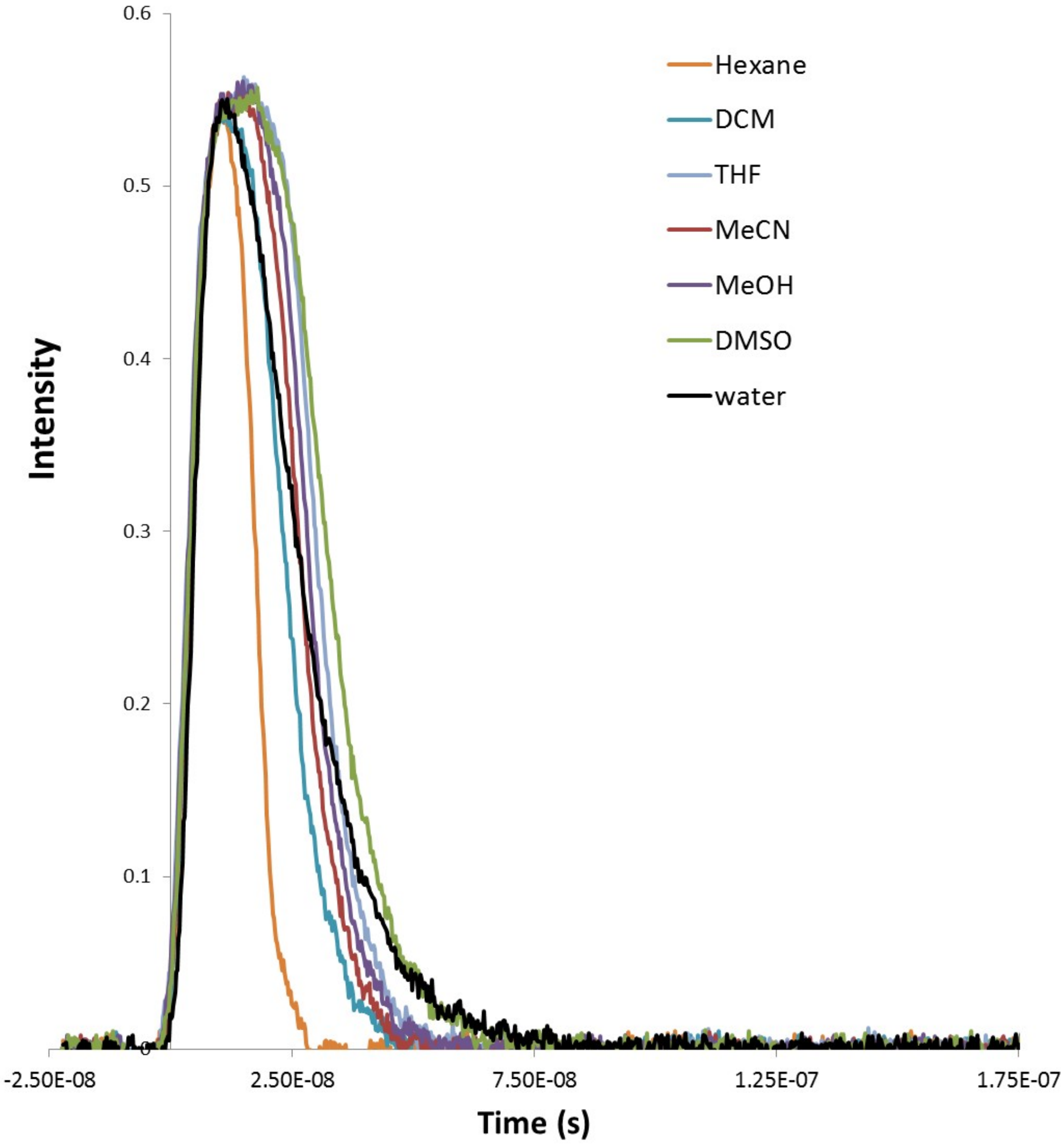

Figure S17. Fluorescence decay of 1,4-ICAN in different solvents.

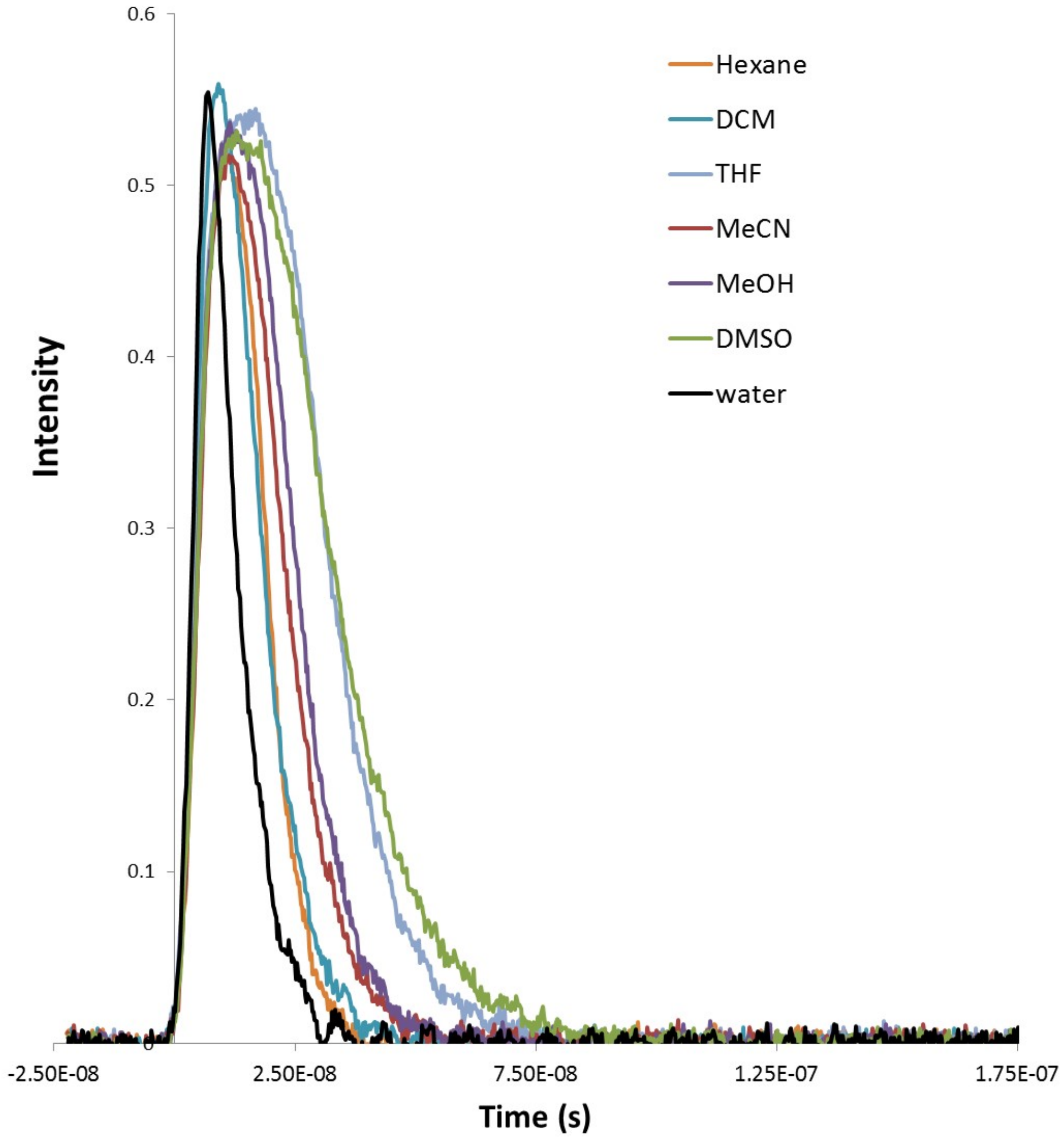

Figure S18. Fluorescence decay of 2,6-ICAN in different solvents.
